# Tree diversity is changing across tropical Andean and Amazonian forests in response to global change

**DOI:** 10.1038/s41559-025-02956-5

**Published:** 2026-01-23

**Authors:** B. Fadrique, F. Costa, F. Cuesta, G. Arellano, L. Cayuela, T. R. Baker, F. C. Draper, A. Esquivel-Muelbert, H. ter Steege, M. Bauters, J. Aguirre-Gutiérrez, Z. Aguirre-Mendoza, M. N. Alexiades, E. Alvarez-Davila, E. Arets, E. Ayala, C. G. A. Aymard, F. Baccaro, S. Báez, C. Baraloto, R. I. Barbosa, P. Barbosa Camargo, J. Barlow, P. E. Barni, J. Barroso, M. Benchimol, A. C. Bennett, E. Berenguer, L. Blanc, D. Bonal, F. Bongers, R. Brienen, F. Brown, M. BT Andrade, B. Burban, R. J. Burnham, J. L. Camargo, S. P. C. Carvalho, C. Castilho, J. Chave, F. Coelho de Souza, J. Comiskey, L. da Costa, R. B. de Lima, E. A. de Oliveira, R. L. C. de Oliveira, R. de Oliveira Perdiz, J. De Rutte, J. del Aguila-Pasquel, G. Derroire, A. Di Fiore, M. Disney, A. Duque, T. Emilio, W. Farfan-Rios, S. Fauset, P. M. Fearnside, K. J. Feeley, T. R. Feldpausch, J. Ferreira, L. Ferreira, G. R. Flores Llampazo, D. Galbraith, K. García-Cabrera, M. García Criado, E. Gloor, J. M. Grandez-Rios, B. Hérault, J. Homeier, E. N. Honorio Coronado, I. Huamantupa-Chuquimaco, W. Huaraca Huasco, Y. T. Huillca-Aedo, Á. Idárraga, O. Jadán-Maza, M. Kalamandeen, T. J. Killeen, S. G. W. Laurance, W. F. Laurance, A. Levesley, W. Lopez, M. J. Macía, W. E. Magnusson, Y. Malhi, A. G. Manzatto, B. S. Marimon, B. H. Marimon Junior, J. A. Martínez-Villa, M. B. Medeiros, K. Melgaço, L. Melo, T. Metzker, A. Monteagudo, P. S. Morandi, J. A. Myers, H. M. Nascimento, R. Nascimento, D. Neill, B. Nieto-Ariza, W. A. Palacios, S. Palacios-Ramos, N. C. Pallqui-Camacho, G. Pardo Molina, J. Peacock, M. A. Peña, R. T. Pennington, M. C. Peñuela, C. A. Peres, Á. J. Pérez, G. C. Pickavance, E. Pinto, J. Pipoly, N. Pitman, A. Prieto, H. Ramírez-Angulo, S. M. Reis, Z. Restrepo, C. Reynel, S. Ribeiro, G. Rivas-Torres, R. Rojas, A. Rudas, N. Salinas, R. P. Salomão, F. Santana, J. Schietti, G. Schwartz, J. Serrano, M. Silman, C. Silva, C. A. Silva, R. C. Silva, R. S. A. Silva, J. Silva-Espejo, M. Silveira, M. F. Simon, Y. C. Soto-Shareva, P. F. Souza, D. Storck-Tonon, J. Stropp, V. Swamy, J. S. Tello, J. Terborgh, R. Thomas, A. Torres-Lezama, J. D. Vale, L. Valenzuela Gamarra, G. van der Heijden, P. van der Hout, P. J. van der Meer, R. Vasquez Martinez, L. Vedovato, H. Verbeeck, I. Vieira, S. A. Vieira, E. Vilanova, B. Vinceti, V. A. Vos, R. Zagt, P. A. Zuidema, O. L. Phillips

**Affiliations:** 1 School of Environmental Sciences, University of Liverpool, Liverpool, UK; 2 School of Geography, University of Leeds, Leeds, UK; 3 Instituto Nacional de Pesquisas da Amazônia, Manaus, Brazil; 4 Global Research and Solutions Center, Universidad San Francisco de Quito, Quito, Ecuador; 5 Ecology and Evolutionary Biology, University of Michigan, Ann Arbor, MI USA; 6 Oikobit, Albuquerque, NM USA; 7 Instituto de Investigación en Cambio Global (IICG-URJC), Universidad Rey Juan Carlos, Móstoles, Spain; 8 Departamento de Biología y Geología, Física y Química Inorgánica, Universidad Rey Juan Carlos, Móstoles, Spain; 9 Department of Plant Sciences, University of Cambridge, Cambridge, UK; 10 School of Geography, Earth and Environmental Sciences, University of Birmingham, Birmingham, UK; 11 Naturalis Biodiversity Center, Leiden, the Netherlands; 12 Quantitative Biodiversity Dynamics, Department of Biology, Utrecht University, Utrecht, the Netherlands; 13 Q-ForestLab, Department of Environment, Ghent University, Ghent, Belgium; 14 Environmental Change Institute, School of Geography and the Environment, University of Oxford, Oxford, UK; 15 Leverhulme Centre for Nature Recovery, University of Oxford, Oxford, UK; 16 Universidad Nacional de Loja, Loja, Ecuador; 17 School of Anthropology and Conservation, University of Kent, Canterbury, UK; 18 Grupo de Investigación en Servicios Ecosistémicos y Cambio Climático, Fundación ConVida, Medellín, Colombia; 19 Wageningen Environmental Research, Wageningen University and Research, Wageningen, the Netherlands; 20 UNELLEZ-Guanare, Programa de Ciencias del Agro y el Mar, Herbario Universitario (PORT), Mesa de Caracas, Venezuela; 21 Jardín Botánico de Bogotá José Celestino Mutis, Bogotá, Colombia; 22 Departamento de Biologia, Universidade Federal do Amazonas, Manaus, Brazil; 23 Departamento de Biología, Escuela Politécnica Nacional del Ecuador, Quito, Ecuador; 24 MODEMAT Foundation for Mathematical Modeling and Education, Quito, Ecuador; 25 Institute of Environment, Florida International University, Miami, FL USA; 26 Instituto Nacional de Pesquisas da Amazônia (INPA - Núcleo de Roraima), Boa Vista, Brazil; 27 Centro de Energia Nuclear na Agricultura, Universidade de São Paulo, Piracicaba, Brazil; 28 Lancaster Environment Centre, Lancaster University, Lancaster, UK; 29 Roraima State University – UERR, Campus Rorainópolis, Rorainópolis, Brazil; 30 Universidade Federal do Acre, Centro Multidisciplinar, Cruzeiro do Sul, Brazil; 31 Applied Ecology and Conservation Lab, Universidade Estadual de Santa Cruz, Ilhéus, Brazil; 32 Forêts et Sociétés, Univ Montpellier, CIRAD, Montpellier, France; 33 INRAE, AgroParisTech, Université de Lorraine, Nancy, France; 34 Forest Ecology and Forest Management group, Wageningen University, Wageningen, the Netherlands; 35 Woodwell Climate Research Center, Falmouth, MA USA; 36 Federal University of Acre, Rio Branco, Brazil; 37 Independent Researcher, Manaus, Brazil; 38 INRAE, UMR ECOFOG, Kourou, French Guiana; 39 University Herbarium, University of Michigan, Ann Arbor, MI USA; 40 Rural University of Rio de Janeiro, Institute of Forestry, Seropedica, Brazil; 41 Centre de Recherche sur la Biodiversité et l’Environnement UMR5300, Université de Toulouse, CNRS, IRD, Toulouse, France; 42 BeZero Carbon, London, UK; 43 National Park Service, Fredericksburg, VA USA; 44 Smithsonian Institution, Washington, DC USA; 45 Instituto de Geociências, Universidade Federal do Pará, Belém, Brazil; 46 Universidade do Estado do Amapá, Amapá, Brazil; 47 Programa de Pós-Graduação em Ecologia e Conservação, Campus de Nova Xavantina, Universidade do Estado de Mato Grosso, Nova Xavantina, Brazil; 48 Universidade Estadual de Roraima, Boa Vista, Brazil; 49 Programa de pós-graduação em Recursos Naturais (PRONAT), Universidade Federal de Roraima, Boa Vista, Brazil; 50 Facultad de Ciencias Forestales, Universidad Nacional Agraria La Molina, Lima, Peru; 51 Universidad Nacional de la Amazonia Peruana, Iquitos, Peru; 52 Instituto de Investigaciones de la Amazonía Peruana, Iquitos, Peru; 53 Department of Forestry, Campus Darcy Ribeiro, University of Brasilia, Brasília, Brazil; 54 CIRAD, UMR EcoFoG (AgroParistech, CNRS, INRAE, Université des Antilles, Université de la Guyane), Kourou, French Guiana; 55 Cirad, UPR Forêts et Sociétés, University of Montpellier, Montpellier, France; 56 Department of Anthropology, The University of Texas at Austin, Austin, TX USA; 57 Estación de Biodiversidad Tiputini, Universidad San Francisco de Quito, Quito, Ecuador; 58 Department of Geography, University College London and NERC National Centre for Earth Observation, London, UK; 59 Departamento de Ciencias Forestales, Universidad Nacional de Colombia Sede Medellín, Medellín, Colombia; 60 Center for Research on Biodiversity Dynamics and Climate Change (CBioClima), Institute of Biosciences, São Paulo State University (UNESP), Rio Claro, Brazil; 61 Andrew Sabin Center for Environment and Sustainability and Department of Biology, Wake Forest University, Winston-Salem, NC USA; 62 Instituto Científico, Universidad Andina del Cusco, Cusco, Peru; 63 School of Geography, Earth and Environmental Sciences, University of Plymouth, Plymouth, UK; 64 Biology Department, University of Miami, Coral Gables, USA; 65 Faculty of Environment, Science and Economy, University of Exeter, Exeter, UK; 66 Embrapa Amazônia Oriental, Belém, Brazil; 67 Museu Paraense Emílio Goeldi, Belém-Pará, Brazil; 68 Universidad Nacional de San Antonio Abad del Cusco, Cusco, Peru; 69 School of Geosciences, The University of Edinburgh, Edinburgh, UK; 70 CREAF, Bellaterra (Cerdanyola del Vallès), Spain; 71 Conservation Ecology, University of Marburg, Marburg, Germany; 72 Resource Management, HAWK University of Applied Sciences and Arts, Goettingen, Germany; 73 Royal Botanic Gardens, Kew, Richmond, London, UK; 74 Herbario Alwyn Gentry, Departamento Académico de Ciencias Básicas, Universidad Nacional Amazónica de Madre de Dios, Madre de Dios, Peru; 75 Centro Ecológico INKAMAZONIA, Valle de Kosñipata, Cusco, Peru; 76 Department of Ecology and Environmental Science, Umeå University, Umeå, Sweden; 77 Asociación para la Investigación Tropical, Cusco, Peru; 78 Jadín Botánico de Medellín, Medellín, Colombia; 79 Grupo de Ecología Forestal y Agroecosistemas, Facultad de Ciencias Agropecuarias, Universidad de Cuenca, Campus Yanuncay, Cuenca, Ecuador; 80 Unique Land Use GmbH, Freiburg im Breisgau, Germany; 81 Museo de Historia Natural Noel Kempff Mercado, Santa Cruz de la Sierra, Bolivia; 82 Centre for Tropical Environmental and Sustainability Science, and College of Science and Engineering, James Cook University, Cairns, Queensland Australia; 83 Coltree, Medellín, Colombia; 84 Universidad Nacional de Colombia, Bogotá, Colombia; 85 Departamento de Biología, Área de Botánica, Universidad Autónoma de Madrid, Madrid, Spain; 86 Centro de Investigación en Biodiversidad y Cambio Global, Universidad Autónoma de Madrid, Madrid, Spain; 87 Departamento de Biologia, Universidade Federal de Rondônia, Porto Velho, Brazil; 88 Université du Quebec a Montreal, Montreal, Canada; 89 Embrapa Recursos Genéticos e Biotecnologia, Parque Estação Biológica, Prédio da Botânica e Ecologia, Brasilia, Brazil; 90 Universidade Federal do Oeste do Pará, Santarém, Brazil; 91 IBAM - Instituto Bem Ambiental, Belo Horizonte, Brazil; 92 Grupo MYR ESG solutions, Belo Horizonte, Brazil; 93 Washington University in St. Louis, St. Louis, MO USA; 94 Coordenação de Biodiversidade, Instituto Nacional de Pesquisas da Amazônia, Manaus, Brazil; 95 Instituto de Ciências Biológicas, Programa de Pós-Graduação em Ecologia, Universidade Federal do Pará, Belem, Brazil; 96 Universidad Estatal Amazónica, Facultad de Ingeniería Ambiental, Puyo, Ecuador; 97 Department of Wetland Ecology, Karlsruhe Institute of Technology, Karlsruhe, Germany; 98 Instituto Nacional de Biodiversidad, Quito, Ecuador; 99 Universidad Técnica del Norte, Ibarra, Ecuador; 100 Systematic and Evolutionary Botany Laboratory, Department of Biology, Ghent University, Gent, Belgium; 101 Instituto de Investigaciones Forestales de la Amazonía, Universidad Autónoma del Beni José Ballivián, Riberalta, Bolivia; 102 Department of Biology, Washington University, St. Louis, MO USA; 103 Geography, College of Life and Environmental Sciences, University of Exeter, Exeter, UK; 104 Grupo de Ecosistemas Tropicales y Cambio Global, Universidad Regional Amazónica Ikiam, Tena, Ecuador; 105 School of Environmental Sciences, University of East Anglia, Norwich, UK; 106 Center for Biodiversity and Global Change, Yale University, New Haven, CT USA; 107 Herbario QCA, Escuela de Ciencias Biológicas, Pontificia Universidad Católica de Ecuador, Quito, Ecuador; 108 Department of Biological Sciences, Auburn University, Auburn, AL USA; 109 Broward County Parks and Recreation, Oakland Park, FL USA; 110 Biological Sciences, Florida Atlantic University, Boca Raton, FL USA; 111 Collections, Conservation and Research, Field Museum of Natural History, Chicago, IL USA; 112 Instituto de Ciencias Naturales, Universidad Nacional de Colombia, Bogota, Colombia; 113 Facultad de Ciencias Forestales y Ambientales, Universidad de los Andes, Merida, Venezuela; 114 Centro de Ciências Biológicas e da Natureza, Universidade Federal do Acre, Rio Branco, Brazil; 115 Servicios Ecosistémicos y Cambio Climático, Fundación Con Vida and Corporación COL-TREE, Medellín, Colombia; 116 Universidad San Francisco de Quito, Estación de Biodiversidad Tiputini, Quito, Ecuador; 117 Wildlife Ecology and Conservation, University of Florida, Gainesville, FL USA; 118 Jardín Botánico de Missouri, Oxapampa, Pasco, Peru; 119 Instituto de Ciencias Naturales, Universidad Nacional de Colombia, Bogotá, Colombia; 120 Department of Science, Chemistry Section, Institute for Nature Earth and Energy, Pontifical Catholic University of Peru, Lima, Peru; 121 Programa de Pós-graduação em Ciências Biológicas, Universidade Federal Rural da Amazônia UFRA/CAPES, Belem, Brazil; 122 Universidade Federal do Amazonas, Manaus, Brazil; 123 Instituto de Pesquisa Ambiental da Amazônia, Brasília, Brazil; 124 Universidade do Estado de Mato Grosso, Caceres, Brazil; 125 Instituto Federal de Educação, Ciência e Tecnologia do Acre, Campus Baixada do Sol, Rio Branco, Brazil; 126 Universidad de La Serena, La Serena, Chile; 127 Instituto de Ecología y Biodiversidad, Santiago, Chile; 128 Department of Biogeography, Trier University, Trier, Germany; 129 Missouri Botanical Garden, St. Louis, MO USA; 130 Department of Biology, University of Florida, Gainesville, FL USA; 131 School of Science and Engineering, James Cook University, Cairns, Queensland Australia; 132 Iwokrama International Centre for Rain Forest Conservation and Development, Georgetown, Guyana; 133 Institute of Research for Forestry Development, Universidad de los Andes, Merida, Venezuela; 134 JDV Ambiental, Toledo, Brazil; 135 Herbario Selva Central HOXA, Oxapampa, Peru; 136 School of Geography, Univeristy of Nottingham, Nottingham, UK; 137 Form International, Hattem, the Netherlands; 138 Van Hall Larenstein University of Applied Sciences, Velp, the Netherlands; 139 Departament of Forest Sciences, University of São Paulo, São Paulo, Brazil; 140 Environmental Studies and Research Center, Universidade Estadual de Campinas, Campinas, Brazil; 141 VERRA, Washington, DC USA; 142 Bioversity International, Rome, Italy; 143 Tropenbos International, Ede, the Netherlands

**Keywords:** Climate-change ecology, Biodiversity, Macroecology

## Abstract

Climate and atmospheric changes are impacting forest function and structure worldwide, but their effects on tropical forest diversity are unclear. Nowhere is the scientific challenge greater than in the Andes and the Amazon, which together include the world’s most diverse forests. Here, using 406 permanent plots spanning four decades of intact lowland and montane forest dynamics, we test for long-term change in species richness and assess the influence of climate and other variables. We show that, at a continental scale, species richness appears stable, but this masks substantial regional variation. Species richness increased in Northern Andean and Western Amazon plots, yet declined in the Central Andes, Guyana Shield and Central-Eastern Amazon. Overall, warmer, drier and more seasonal forests lost species, while those at higher elevations, in less fragmented areas and with faster rates of tree turnover experienced increases. Region-specific drivers, particularly precipitation seasonality and demographic factors, modulated these trends. The results highlight the diverse ways in which Amazon–Andes forests are changing and underscore the critical need to preserve large-scale ecosystem integrity to maintain local tree diversity. By doing so, Northern Andean forests in particular could serve as an important refuge for species increasingly displaced by climate change.

## Abstract (web summary)

This study examines long-term changes in species richness across tropical forests in the Andes and Amazon. Hotter, drier and more seasonal forests in the eastern and southern Amazon are losing species, while Northern Andean forests are accumulating species, acting as a refuge for climate-displaced species.

## Main

The Andes and the Amazon are crucial for carbon storage, biodiversity conservation and climate regulation^1–5^. However, climate change and land-use change are threatening the stability of these ecosystems and the services they provide^5–10^. Over recent decades, temperatures have increased in this region, precipitation patterns have become more extreme and variable, deforestation has expanded and forest fires have become more frequent^7,11–15^. Under these increasingly stressful conditions, plant species have two feasible short-term responses to survive: (1) migrate—shift their distribution range in response to changing environmental conditions, or (2) acclimate—utilize their physiological tolerance to maintain function under the new conditions. If species do not manage to migrate or acclimate, their populations will decrease and eventually they may go extinct^16^.

The response of plant species to climate change could lead to changes in forest structure, composition, diversity and species richness at the local scale^17–19^. The Andes and other tropical mountains are undergoing a process of thermophilization, where higher-elevation forests are incorporating new lower-elevation species that expand their ranges upslope, and current low-elevation species are increasing in relative abundance^20–23^. However, lower-elevation forests face the possibility of biotic attrition (a net loss of species), as there is no species pool from even hotter areas able to migrate and fill the new thermal niches^24–26^. While the wet tropics have been suggested to have the highest rates of plant extinction, based on literature reviews^27^ and model predictions^28^, we do not know whether this translates into consistent losses of local richness within the different regions in the Andes–Amazon area.

Despite widespread threats across the Andes–Amazon area, climate change and other large-scale disturbances are not distributed evenly across space^7,9,29^. Moreover, geographical features—such as increased topographical variation, which may provide a potential advantage for species persistence by offering more suitable environmental conditions—are also unevenly distributed^30,31^.

At the local scale, stressors, such as increasing temperatures and declining rainfall, have been related to mortality-driven compositional shifts, particularly in steep elevational gradients^21,30,32,33^. Baseline temperature and precipitation regimes have also been shown to relate to the probability of plant species suffering thermal or drought damage^34,35^. Fragmented areas are also vulnerable to diversity losses, while increasing fire frequency reduces regeneration and species richness^13,36,37^. However, although several mechanisms have been shown to drive changes in (neo)tropical forest diversity, most studies so far have been limited to local or regional scales and/or lack long-term assessments of tree richness and diversity at consistently monitored sites. Indeed, long-term compositional changes have often been estimated using modelling approaches and have rarely been addressed using field data (but see refs. ^33,38^).

Here we use 406 long-term floristic plots, measured for different time periods since 1971 across 10 countries in South America to estimate the magnitude and direction of tree richness change through time and to identify their drivers. Across this vast space, ranging from −17 to 8.5 latitudinal degrees and −80 to −47 longitudinal degrees, we explore the change in richness through time for the combined area and independently for each of six predefined regions (based on their geomorphological and biogeographical history and contemporaneous geoecological features), as we hypothesize that different regions are responding in different ways, forced by different drivers. Using consistent methods to identify the spatial distribution of diversity change and the factors that contribute to it at a larger scale is crucial to understanding the current status of the Amazon and Andean forests, predicting future patterns and informing conservation efforts. With this comprehensive plot compilation and a set of climatic and structural variables, we intend to answer the following questions. First, using the complete dataset, we ask: (1) How is tree species richness changing across the Andes–Amazon area? Is there an overall decline? and (2) How are changes in richness related to baseline climate, climate change, landscape context and forest structure?

We predict an overall stability of richness, with local increases and declines balancing each other out. However, we expect the change in richness to be associated with several large-scale variables. In particular, we expect a more pronounced decrease in richness in warmer, drier forests at lower elevations given the thermophilization trend where species are ‘migrating’ towards higher elevations that usually tend to be colder and wetter. Similarly, we predict a richness decline in forests that are becoming warmer or drier as dealing with this climate becomes physiologically more challenging. We also expect a decrease in species richness in forests with high fragmentation due to the reduced source of colonizers and habitat connectivity. A summary of all the predictors tested and our hypothesized relationships with species richness change is presented in Table 1.

**Table 1:** Predictors included in the study, along with their acronyms, units, time frame used for calculation, brief description and hypothesized relationship with species richness change

	Predictor	Units	Time frame	Description	Hypotheses
Baseline climate	Maximum temperature	°C	1979 to final census	TerraClimate. Mean annual maximum temperature (highest maximum monthly temperature of the year).	Negative relationship. Across temperature gradients, warmer forests may be more affected by biotic attrition, as they are closer to the trees’ physiological limits, while cooler forests can incorporate lower-elevation species^26^.
	Annual precipitation	mm	1979 to final census	TerraClimate. Mean annual cumulative precipitation.	Positive relationship. Drier forests may be more affected by biotic attrition, as they are closer to the trees’ physiological limits and they could present more hydraulic stress, which should be more challenging for new species to grow^76^.
	Precipitation seasonality	CV	1979 to final census	TerraClimate. Mean annual standard deviation of monthly precipitation as a percentage of the mean.	Negative relationship. Higher precipitation seasonality is related to lower tree diversity, so we expect highly seasonal forests to be more prone to species loss^38,77,78^.
Climate change	Temperature change	°C yr^−1^	1979 to final census	TerraClimate. Annual change in the mean annual maximum temperature. Calculated as the linear model regression coefficient.	Negative relationship. Faster-warming forests may be losing more species than slower-warming or cooling forests owing to the challenges of dealing with a temperature higher than their optimum^79^.
	Precipitation change	mm yr^−1^	1979 to final census	TerraClimate. Annual change in the mean annual precipitation. Calculated as the linear model regression coefficient.	Positive relationship. Forests becoming drier are expected to present harder conditions for species and even prompt some local extinctions. Forests becoming wetter could relieve the hydraulic stress of some species and encourage recruitment^33,80^.
	Precipitation seasonality change	CV yr^−1^	1979 to final census	TerraClimate. Annual change in precipitation seasonality. Calculated as the linear model regression coefficient.	Negative relationship. In line with precipitation seasonality, more extreme seasonality may be related to a decline in species richness, while more stable precipitation seasonality would maintain species richness^38,81^.
Landscape context	Landscape integrity	%	2015	GFCC Tree Cover Multi-Year Global 30-m raster, aggregated to 120-m pixels in GEE. Mean percentage tree-covered pixel area in 2015 in a 50-km radius from each plot.	Positive relationship. Forests surrounded by more vegetated areas should have a larger pool of species within dispersal distance to potentially recruit^82,83^.
	Elevation	m a.s.l.		SRTM 90-m resolution.	Positive relationship. Lower-elevation species could recruit at higher elevations to maintain their optimum temperature requirements. Together with some extinction lag, this could increase richness in the higher elevations^44^.
Structure	Stem abundance change	% yr^−1^	Census interval	Difference in the number of individuals between final and initial census divided by the initial number of individuals and multiplied by 100, then divided by the time between censuses.	Positive relationship. A higher number of individuals increases the likelihood of encountering new species, thereby raising local species richness^84^.
	Mortality rate	% yr^−1^	Census interval	Logarithm of initial stems minus logarithm of surviving stems divided by time between census and multiplied by 100 and by the census interval to the 0.8 power. Sheil and May 1996^72^ equation and Lewis et al. 2004^73^ correction.	Mixed relationship. Faster mortality rates indicate newly opened areas that are susceptible to colonization. When coupled with rapid recruitment, this can increase the likelihood of encountering new species. Meanwhile, higher mortality reduces overall tree abundance, which can decrease species richness^85^.
Sampling	Identification effort change	%	Census interval	Change in the percentage of individuals per plot (from final to initial) that were identified to species level.	Positive relationship. A greater number of identified individuals increases the likelihood of recording new species, thereby enhancing local richness.
	Time frame	years	Census interval	Years between initial and final census.	No relationship.

Then, analysing each of six predefined regions separately, we ask: (3) Does the change in tree richness exhibit the same trend in the different regions of the Andes–Amazon area? and (4) Which of the selected predictors explain the change in species richness for each region?

We expect to find a longitudinal gradient in diversity change across the six Andes–Amazon regions driven by the most pressing stressors in each region. In particular, we hypothesize (a) an increase in richness in the Andes as a consequence of thermophilization and a decrease of richness in the Amazon, particularly in the drier and warmer Central-Eastern regions (Guyana Shield, Central-Eastern Amazon and Southern Amazon), due to biotic attrition; (b) temperature will thus be a crucial factor in the Andean trends, while precipitation could be more important in the Amazon; and (c) landscape integrity will have an important role in the more degraded Southern and Central-Eastern Amazon regions

## Results

### No overall change in the richness of the Andes–Amazon area

Half of our plots (203) declined in richness, and 146 increased. Richness change varied widely across plots (range −1.95% to +3.3% per year) but had no consistent direction at the Andes–Amazon scale (bootstrapped mean richness change 0.036, mean confidence interval (CI) −0.09 to 0.16, mean *t* statistic 0.579, mean *P* value 0.56, degrees of freedom 179) (Supplementary Fig. 1).

**Fig. 1:**
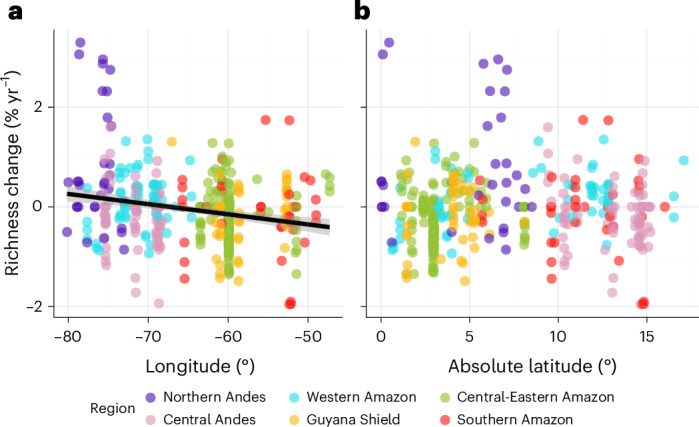
Richness change at sampling location. **a**,**b**, Relationship between plot location and richness change per plot: longitude in decimal degrees (**a**) and absolute latitude in decimal degrees (**b**). Each point represents a plot, and its colour corresponds to the region. The solid line represents statistically significant (*P* < 0.001) linear regression. The shaded ribbon represents the 95% CI. Source data

We found a negative relationship between richness change and longitude (slope −2.39, adjusted *R*^2^ = 0.047, *P* < 0.001). At −64.5°, which coincides broadly with the transition between the Eastern and Western Amazon, the change in richness shifts from positive (West) to negative (East) values (Fig. 1). There was no significant relationship with latitude.

### Richness change drivers at the Andes–Amazon scale

**Fig. 2:**
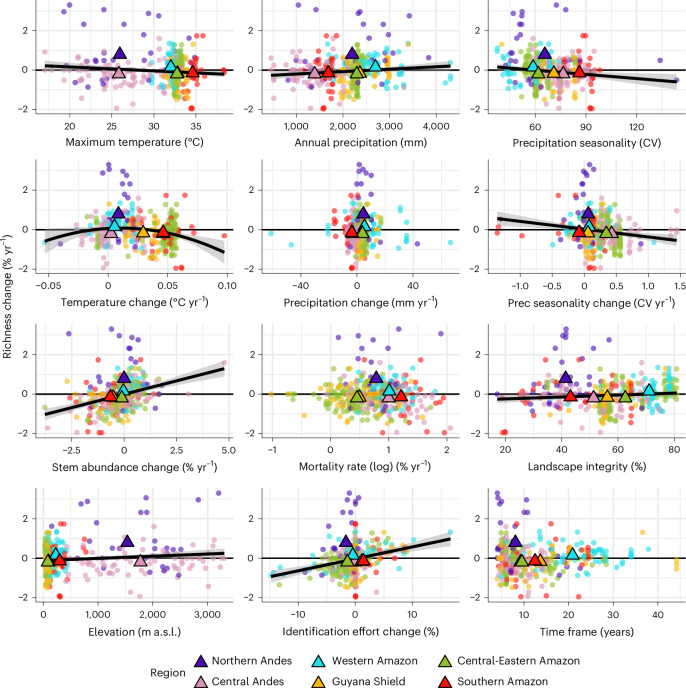
Richness change and predictors relationships across the Andes and Amazon. Bivariate regression between richness change (% yr^−1^) and the different predictors. Colours indicate regions. Points are individual plots (*n* = 406), and triangles are regional means (*n* = 6). Solid lines represent statistically significant regressions (*P* ≤ 0.05). Shaded ribbons around lines represent 95% CI. For extended statistical results, see Supplementary Table 1. Source data

In the bivariate regressions with the complete dataset, we found that maximum temperature, precipitation seasonality and precipitation seasonality change had significant negative relationships with richness change (Fig. 2 and Supplementary Table 1; see predictor description in Table 1). Temperature change exhibited a hump-shaped relationship with richness, decreasing slightly where temperatures cooled and more markedly where warming was faster. Annual precipitation, stem abundance change, landscape integrity, elevation and identification effort change had positive significant relationships (Fig. 2). The bootstrapped regressions corrected for spatial bias in plot location supported the representativity of the overall trends found as slope direction and significance coincided for most of the variables (Supplementary Table 2 and Supplementary Fig. 2). The regression with annual precipitation, although always positive, was on average not significant in the bias-corrected analysis, and the one with landscape integrity was typically positive but not significant, probably because of the confounding effect of decreasing tree cover with elevation in the Andes.

**Extended Data Fig. 1:**
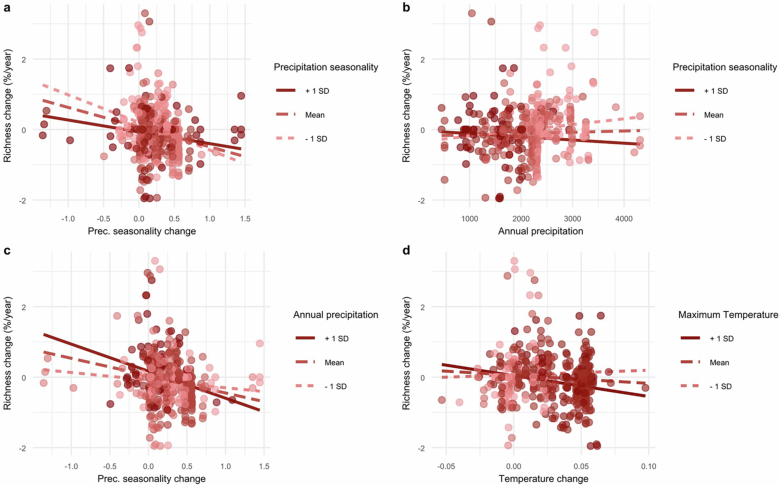
Regression between interacting climate variables and species richness change. Interaction plots showing the results for the regression of the interaction between **a**) Precipitation seasonality and Precipitation seasonality change, **b**) annual precipitation and precipitation seasonality, **c**) precipitation seasonality change and annual precipitation, and **d**) temperature change and maximum temperature (regression results in Table SI3) for the whole dataset (n = 406). Line and point colours indicate the second predictor categorised into three groups (mean, +1 SD, −1 SD). Source data

When predicting richness change, we observed significant interactions between precipitation seasonality and its change, precipitation seasonality and annual precipitation, and annual precipitation and precipitation seasonality change (Extended Data Fig. 1 and Supplementary Table 3). Species richness declined with increasing precipitation seasonality, but this decline was steeper for less seasonal forests. Species richness in less seasonal forests increased with annual precipitation. We found marginal support for an interaction between the temperature variables, suggesting that warmer forests experiencing further warming lost more species, whereas cooler forests even showed a slight increase in richness.

### Andes–Amazon regions experienced different trends of richness change

**Fig. 3:**
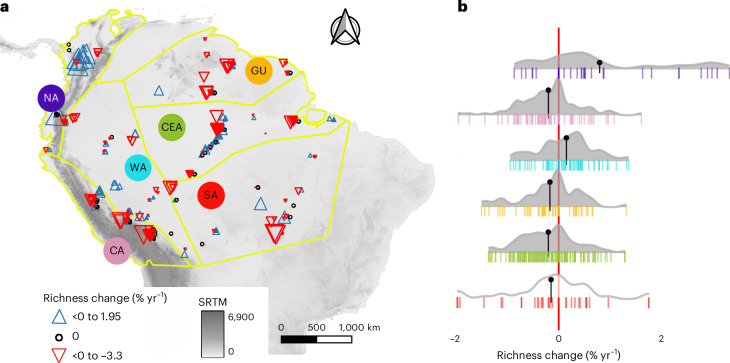
Forest plot and regional changes in richness. **a**, Map showing the distribution of the 406 plots (arrowhead symbols located at plot coordinates) in the six regions (NA, Northern Andes; CA, Central Andes; WA, Western Amazon; GU, Guyana Shield; CEA, Central-Eastern Amazon; SA, Southern Amazon). Symbol colour and angle represent richness change direction, and symbol size is proportional to the magnitude of change for each plot. Black circles represent no net change. Background SRTM represents elevation in m a.s.l. (Table 1). **b**, Richness change (% yr^−1^) per region expressed as proportional change in relation to the initial census. Tick marks represent individual plots. The shaded area represents the density distribution of the plots. Shade colour indicates a significant difference from zero using a two-sided *t*-test (grey, *P* ≤ 0.05; white, *P* > 0.05; NA, *P* < 0.0001; CA, *P* < 0.0001; WA, *P* = 0.003; GU, *P* = 0.004; CEA, *P* < 0.0001; SA, *P* = 0.29). For extended results, see Supplementary Table 4. Source data

Richness change was directional in five of the six regions (Fig. 3). Species richness significantly increased in the Northern Andes and Western Amazon, while the Central Andes, Central-Eastern Amazon and Guyana Shield experienced significant declines. Although the Southern Amazon did not show a significant trend, the mean change was negative and included some of the most extreme negative values. The direction of these changes coincided across the other diversity indices tested (Supplementary Table 4), although the significance of the change was more variable because different indices reflect slightly different aspects of diversity change (Supplementary Note 1).

### Regional trends have different explanatory predictors

**Fig. 4:**
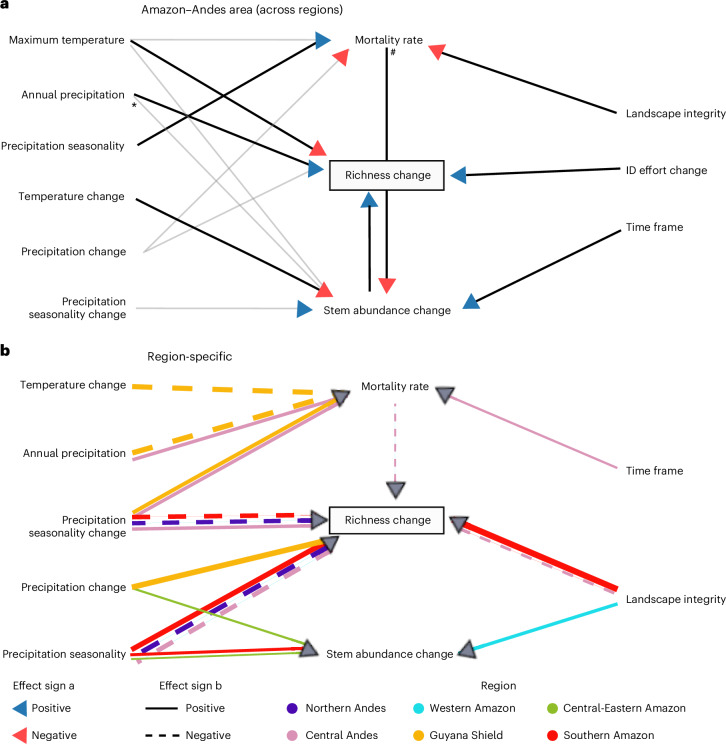
Results from the multigroup SEM analysis. Diagram illustrating the relationships between the independent variables in the model, with richness change as the final response variable, and stem abundance change and mortality rate as intermediate response variables that may also influence richness change. Both panels are part of the same SEM, but for easier interpretation, they show general and region-specific relationships separately. **a**, Significant relationships constrained across the study area, with arrowhead colour indicating negative or positive effects. The effect of annual precipitation on stem abundance change (marked by asterisk) is constrained to 0. The effect of mortality rate on stem abundance change (marked by hash sign) is positive and significant across regions but not constrained. Non-significant constrained relationships are shown in grey. **b**, Significant relationships in specific regions, with arrow colour indicating the region, width representing the standardized effect size (in mm × 2) and stroke style denoting the effect sign (solid, positive; dashed, negative). For standardized effect sizes of all variables, see Supplementary Table 5. Source data

We used a multigroup piecewise structural equation model (SEM) analysis to identify the relationship between the predictor variables and the richness change directly and indirectly. This SEM (Fig. 4) showed a good fit to the data (Fisher’s *C* = 4.232, *P* = 0.375). The individual *R*^2^ for the component models were 0.18 (mortality), 0.27 (stem abundance change) and 0.30 (species richness change). Complete model results are presented in Supplementary Table 5.

Many of the relationships between climate and environmental variables with stem abundance change and mortality rate were constrained (indicating a similar effect) across regions (Fig. 4 and Supplementary Fig. 3). For stem abundance change, five out of eight variables were constrained, with two of these being significant; for mortality, four out of the eight variables were constrained, with two of them significant. For richness change, 5 out of 11 variables were constrained, with 4 being significant.

**Fig. 5:**
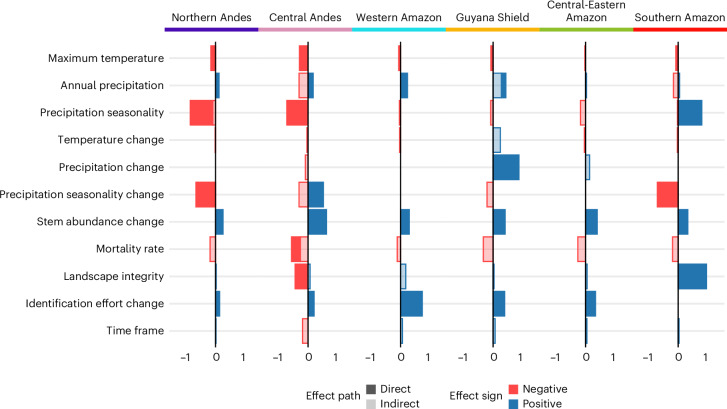
Standardized effect of each predictor on richness change from the multigroup SEM analysis. Only significant (*P* ≤ 0.05) direct effects are shown. Indirect effects were calculated by multiplying the significant (*P* ≤ 0.05) standardized coefficients within each of the possible three indirect pathways (via stem abundance change, via mortality rate, and via mortality rate × stem abundance change) and then adding them. The transparency of the bar represents the effect path, with direct effects being opaque and indirect effects transparent. The bar colour indicates the sign of the effect (red is negative, blue is positive). Region colours are shown in the top line for coherence with Fig. 4. For extended results, including specific *P* values, see Supplementary Tables 5 and 6. For standardized effects of each predictor variable for each region, see Supplementary Table 7. Source data

Regarding the intermediate factors mediating indirect effects, mortality rate had a significant negative effect on richness in the Central Andes, and stem abundance change had a significant positive effect on richness change in all regions. We computed the indirect effects that the predictors had on richness change mediated by the structural variables when each path coefficient was significant (Fig. 5 and Supplementary Tables 6 and 7).

Maximum temperature had a total negative effect on richness across regions, while precipitation had a general positive effect. Precipitation seasonality had a strong negative effect in the Andes but positive in the Southern Amazon. Temperature change had a very small negative effect in the Central Andes, Western Amazon and Central-Eastern Amazon. Precipitation change had a large positive effect in the Guyana Shield. Precipitation seasonality change was variable, having a large negative effect in the Northern Andes and Southern Amazon but a positive effect in the Central Andes. Stem abundance change had a positive effect in all regions, while mortality had a negative effect. Landscape integrity had a strong positive effect in the Southern Amazon, weaker positive effects in other regions and a negative effect in the Central Andes. Change in identification effort had a positive direct effect in all regions except the Southern Amazon, while the time frame had very small positive effects in five regions and a negative effect in one region.

## Discussion

### No apparent overall change in tree richness of the Andes–Amazon area

We found no overall trend in species richness change across 406 forest-dynamics plots distributed across the tropical Andes and the Amazon. However, this large-scale result masks important regional variations, with richness increasing in the Northern Andes and Western Amazon, while decreasing in the Central Andes, Central-Eastern Amazon and Guyana Shield. This masking or obscuring issue has been raised for global estimations of diversity change based on local trends, and some even question the relevance of these large-scale averages^39–41^. In any case, the absence of a significant overall trend in richness change may also indicate a temporary disequilibrium between current environmental conditions and large-scale vegetation responses^42^, which should not be misinterpreted as resilience. Lag effects could occur on the leading edge, where trees slowly colonize newly suitable habitats, delaying potential richness gains. Alternatively, lags at the trailing edge could indicate a temporary persistence of species, artificially inflating current richness estimates^42^. Lowland areas of the Amazonia are expected to experience greater lags due to the long migration distances required to remain at equilibrium with their optimal conditions^43^. By contrast, mountain regions are thought to have an extinction debt, allowing temporary species accumulation^44,45^.

Across the Amazon, current tree diversity patterns are largely shaped by seasonality, with higher diversity found in the wet, aseasonal forests of the Western Amazon and lower diversity in the drier, seasonal forests of the Eastern regions^46^. Our findings on species richness change align with this longitudinal gradient, revealing negative trends in the Eastern regions and positive trends in the Western regions. We first discuss these large-scale patterns, followed by the regional findings that help explain these trends.

### Climate stress versus structural resilience

Hotter, drier and more seasonal forests and those getting warmer and more seasonal are losing species, but forests with more trees and higher landscape integrity are gaining them. Over the past 40 years, more than 90% of our plots (368/406) have experienced warming with a mean rate of 0.028 ± 0.018 °C per year (321/406 during the individual monitoring periods). Faster-warming forests in the Central-Eastern and Southern Amazon (0.05 ± 0.02 °C per year) are losing species at a higher rate than forests experiencing more moderate warming. In addition, forests in warmer areas within the Andes–Amazon area are also losing more species (Fig. 2). This pattern reflects the contrasting conditions and biotic responses of the Andes and Amazon forests and, supported by the higher rate of species accumulation with increasing elevation (Fig. 2), provides further evidence for thermophilization in the region^21,30,47^. This phenomenon is also supported by the temperature interaction, where the impact of heating in driving species richness loss depends on the baseline temperature, with hotter forests being more sensitive to a given rate of heating. Nevertheless, most of the forests in the Central Andes that experienced slight cooling (50/76) also showed negative trends in species richness (29/50), probably influenced by a decline in precipitation and an increase in seasonality in all of these plots (29/29). This trend indicates that precipitation change modulates richness responses to temperature.

Rainfall declined in 39% of plots, but its influence was minor relative to that of precipitation seasonality, which increased in 88% of the plots. Forests that are more seasonal—and especially those becoming more seasonal—showed declines in species richness^17^, with the strongest negative effects in currently less seasonal or wetter forests (that is, higher annual precipitation; Extended Data Fig. 1). These results agreed with findings from the Andean mountain tops, where seasonality across the latitudinal gradient is strongly linked to richness changes, with more aseasonal peaks near the Equator showing richness gains^38^. While we did not find a latitudinal trend across the study area (Fig. 1), we observed differences between the Northern and Central Andes, which we discuss in detail below.

The more individuals recorded in a census, the larger the gain in the number of species (Fig. 2), as expected because more species from the regional pool have a chance to recruit. This pattern extends beyond individual plots, as forests in less fragmented landscapes (higher landscape integrity), surrounded by more contiguous forest, are more likely to show increases in species richness. By contrast, forests that become more isolated from surrounding fragments tend to experience a decline in species richness^36,48,49^.

### Diverse regional patterns of richness change and diverse drivers

The Western Amazon and Northern Andes are gaining species, while the Central Andes, Guyana Shield and Central-Eastern Amazon are experiencing species loss (Fig. 3). According to the SEM, the processes driving changes in tree density and mortality rates are similar across the Andes–Amazon area (Fig. 4). Generally, mortality rates rose in more seasonal and fragmented forests, while stem abundance declined in warming forests and in forests experiencing higher mortality rates. The relationship between richness change and environmental variables revealed many region-specific drivers, with some variables having opposite effects in different regions, highlighting the context-dependent processes in our vast study area.

The relationship between stem abundance change and richness change was positive across regions. This means that a greater decline in the number of individuals in a plot (in proportion to the initial number) was associated with a more negative change in species richness, and vice versa. Changes in individual abundance are crucial for enabling compositional change, as more recruits increase the likelihood of detecting new species from the local pool^50^. However, the entry of new species does not necessarily imply a shift in composition outside the existing regional pool, and species loss could reflect local extinctions or shifts within the same pool. Further analysis is needed to determine whether these species are new or are part of the regional pool. All regions showed a negative trend in stem abundance, with the Eastern Amazon (Guyana Shield, Central-Eastern and Southern Amazon) experiencing sharper declines than the Western Amazon and the Andes, which showed higher variability. This is contrary to the results of previous research showing an increase in stem density across 50 Amazonian plots from 1979 to 2002^51^. Although this discrepancy may simply reflect the differing sample sizes and geographical extents of the studies, it could also indicate a recent change in the stem density trend driven by rising temperatures.

Mortality directly affected only the Central Andes, with its effects on other regions mediated through stem abundance change. Thus, the hypothesized disturbance effect of mortality in promoting the colonization of new species is probably limited to the Central Andes.

**Extended Data Fig. 2:**
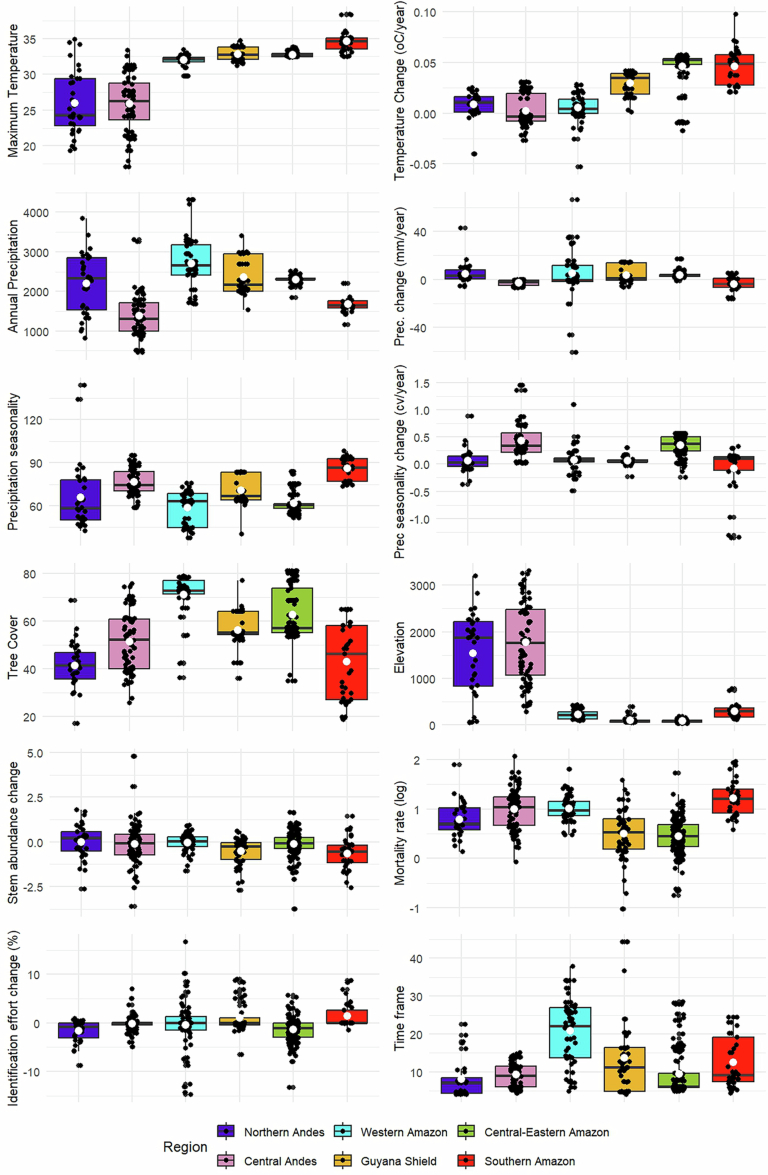
Regional predictors. Distribution of values for each of the predictors for each region. Error bars represent the most extreme data points, which are no more than 1.5 times quantiles 1 and 3 of the data (represented by the box limits). Box crossline represents the median. White points denote mean values. Number of plots per region: NA:32, CA:76, WA:63, GS:53, CEA:145, SA:37. Source data

Across regions, warmer and drier areas are linked to lower rates of richness change. Regional temperature gradients, particularly elevation gradients in the Andes, play a crucial role in richness change. We found an increase in richness in the Northern Andes, which agrees with the reported compositional change caused by the thermophilization process in the area^21,30,52^ and with research showing a warming-related increase in mountain-top diversity^38,44^. The encroachment of lower-elevation, warm-adapted species, which would initially be rare in the community, would lead to a potentially temporary increase in the number of species supported by the extinction lag of cold-adapted species that cannot tolerate the new conditions and will eventually become locally extinct^53,54^. We expected that both Andean regions would share the same pattern; however, the Central Andes showed a decline in richness. Our results suggest that the faster-warming Northern Andes region^14^ could be more suitable for range shifts than the more moderate—and even cooling—Central Andes (Extended Data Fig. 2). The most important factor determining the richness change in the Northern and Central Andean regions was change in precipitation seasonality, having a negative effect in the Northern Andes and a positive effect in the Central Andes. Across the Andes, precipitation and its seasonality are highly variable, being affected by local orography, orientation and cloud cover^14^; however, on average, the Central Andes are drier and more seasonal than the Northern Andes, and they are also becoming drier and more seasonal at a faster rate (Extended Data Fig. 2 and Supplementary Table 8). We hypothesize that migrating lower-elevation species, particularly those distributed in the Western Amazon, are more likely to succeed expanding into higher elevations of the wetter and less seasonal Northern Andes than in the Central Andes. The Central Andes probably pose a greater barrier from water-related physiological stress (particularly when compared with the Western Amazon) than the Northern Andes. Furthermore, the negative relationship between richness and landscape integrity in the Central Andes probably results from the confounding effect of decreasing tree cover with elevation.

The Western and Central-Eastern Amazon presented a very similar breakdown of driver effects. In both regions, changes in stem abundance were the primary ecological drivers, with minor indirect effects from climate variables, largely mediated by the change in stem abundance. In these regions, forests that are warmer, drier or becoming warmer or drier exhibited declining richness, as these conditions reduce the number of individuals. The Central-Eastern Amazon is drier and is warming faster than the Western Amazon, which could explain the overall richness decrease in the Central-Eastern Amazon as opposed to the increase in the wetter Western Amazon.

In the Southern Amazon, where there was no significant trend in richness change, and in the Guyana Shield, which showed a negative trend, precipitation and its seasonality played predominant roles. In the Guyana Shield, dry forests—and particularly those becoming drier—experienced the greatest species losses. In the Southern Amazon, which is highly seasonal, there is evidence that forests that were more seasonal at baseline tended to gain species; however, increases in precipitation seasonality were associated with richness declines. Nevertheless, in the Southern Amazon (the area with some of the most fragmented forests), landscape integrity exerted the strongest direct effect on richness change: forests embedded within larger, contiguous forested areas tended to gain species, whereas more fragmented forests tended to lose them.

Landscape integrity also had a negative relationship with mortality rate across all regions, indicating that higher landscape integrity supports tree survival, thereby increasing tree abundance, which, in turn, positively impacts richness. This agrees with previous findings on the damaging effects of deforestation and/or degradation in surrounding forests, underscoring the importance of preventing forest fragmentation to support biodiversity conservation^55^. It also highlights the conservation priority of the Western Amazon–Northern Andes corridor, which appears to be the most feasible pathway for range shifts that could support species persistence.

This study provides a comprehensive assessment of tree richness change in the Andes–Amazon forests using long-term field data. However, we acknowledge that we are working in one of the most diverse and dynamic areas of the planet^56^, and, as such, there are limitations to our analyses. First, the dataset lacks a historical baseline, so initial conditions may be influenced by uncertain processes^39^. To minimize bias, we used strict plot selection criteria, excluding plots with any sign of fire or large disturbances and directly including identification effort change and time between censuses as predictors in our analyses. The change in identification effort positively influenced richness change across regions: as more individuals are identified, we encounter more species. Monitoring time had only a small effect on species richness change, where shorter intervals capture more noise relative to the signal than longer intervals.

Second, climatic and environmental data extracted from global databases add uncertainty, especially in topographically complex areas like the Andes. At an even finer scale, it is impossible to know the real climate experienced on the forest floor by each individual tree; further investment in microclimate monitoring in these structurally complex forests is crucial to improve our understanding of climate change effects. Third, we are including only trees with a diameter at breast height (DBH) greater than 10 cm and are ignoring the potential contribution of smaller size classes to changes in diversity. Finally, there are multiple factors not accounted for in the study that can have important roles in diversity trends. For example, it was beyond the scope of this study to evaluate the roles of past forest history, including Indigenous management, in current richness trends, nor did we evaluate the potential role of biotic pressures (for example, herbivory and pathogens), nor that of conservation efforts and compensation mechanisms, including carbon and biodiversity benefits. Further research should address more complex compositional questions, such as evaluating the taxonomic and functional identities of species being lost or recruited, and whether this indicates that the Andes–Amazon is undergoing taxonomic homogenization, functional homogenization or both.

In conclusion, across the study area, hot, dry and seasonal forests and those becoming warmer and more seasonal are losing species, while forests with higher tree density and higher landscape integrity are gaining them. Our large-scale findings emphasize the critical role of temperature and temperature change in shaping tree richness in the Andes–Amazon area. However, at the regional level, precipitation and its shifts in distribution and annual amounts play more important and region-specific roles, outweighing the influence of temperature^57^.

This study highlights the uneven impact of changing environmental conditions on tree diversity across different tropical forests, as well as the varied importance of climate and environmental variables across the different regions and scales. Our results underscore the key role of the Northern Andes as a refuge for tree species facing increasingly unsuitable climatic conditions in the Amazon. Finally, our findings highlight the tight relationship between preserving tree abundance and preserving diversity, emphasizing the enormous threat posed by land-use change, which indiscriminately reduces both tree abundance and regional species diversity.

## Methods

### Forest monitoring plots

We combined permanent plot data from ForestPlots.net^58^ (https://forestnet.com/) and from the Madidi project (https://madidiproject.weebly.com). Plot establishment and resurveys were performed by well-trained field teams that followed a detailed protocol that included geolocating plot boundaries, marking subcorners with permanent polyvinyl chloride tubes, taking tree subplot and coordinate data, tagging trees with numbered aluminium tags, and noting and painting the point of measurement. Post-field quality control was carried out by database managers and the field team leader. We selected all plots within the study area (Andean or Amazonian country in areas lower than 4,000 m above sea level (a.s.l.)) that had been censused at least twice. We did not include plots located in the Chocó and the Northern Venezuela regions because of insufficient sample sizes to represent these areas. To avoid the confounding effects of successional trends on diversity change, we included only plots in forests that were undisturbed or had experienced disturbance at least 50 years prior (identified as equivalent to long-term successional forest). For the same reason, we excluded plots that had been recorded on ForestPlots.net as swamp or seasonally flooded forests or as having a history of fire or of large disturbances. We also excluded plots that had been flagged for having taxonomic identification issues.

We obtained curated datasets for each census and plot. For each plot, we selected the first and the last census. Hereafter, we refer to these two censuses as ‘initial’ and ‘final’. We ensured that plot area and location exactly matched on both censuses and that the plot sampling strategy was standardized across time. For instance, we excluded palms when they were not measured in every census.

To standardize methodologies, we removed from the dataset subplots (delimited sections within a plot) in which the protocol required a minimum tree DBH greater than 10 cm for inclusion. We also removed all individuals smaller than 10 cm DBH and those belonging to the families Cyclanthaceae and Araceae. Species taxonomic identification was carried out in the field and in the herbaria where reference collections with vouchers are deposited. Any change in an individual’s identification was applied across all censuses. To minimize the impact of the change in identification effort (the proportion of individuals identified to species level) between censuses, we restricted our analyses to plots that (1) had more than 50% of the tree individuals identified to species level in the initial census, (2) had a difference in the proportion of identified individuals between first and last census smaller than 10% and (3) had at least 50% of the recruits in the final census identified to species (when there were more than 20 recruits). In some instances, this meant using the next-to-last census within the plot as the final census. The change in the percentage of individuals identified to species level is used in the model as a predictor to account for the potential confounding effect of this factor.

We used the taxonomic name resolution service (TNRS) tool^59^ (https://tnrs.biendata.org) and R package^60^ to standardize species names. We manually verified matches with an overall score <0.9, and ‘unclear’ and ‘not found’ matches. We looked for potential explanations such as spelling errors in the Tropicos (https://www.tropicos.org) and WFO (https://wfoplantlist.org/plant-list) lists, and we either manually modified the accepted name for these species or used only their genus ID if there was no clear option. As the treatment of morphospecies was not curated or standardized across the dataset, we converted any morphospecies codes into ‘Genus indet’ format to group morphospecies into genera across the dataset. See the ‘Unidentified species and morphospecies’ section for an overview of the process of integrating morphospecies into the analyses.

Given that the plot size varied widely, we grouped plots that were less than 0.5 ha in area if they had other plots within a 7-km radius with no indication of large differences (that is, similar elevation, forest type, soil classification and so on). For quantitative metadata values, such as the time between censuses, we used the mean. We will refer to these plot groupings as ‘plots’, given that they are treated as a single unit. We also reduced the size of our biggest plots (plot areas of 25 and 9 ha) by selecting two 1-ha subplots on opposite corners and treating them independently. We then eliminated plots that had intervals of less than 4 years between the two selected censuses, because we considered this time elapsed to be too short to provide mid-to-long-term diversity change information. The time elapsed between the initial and final censuses was used in the model as a predictor to account for its potential confounding effect.

Finally, after preliminary exploration of plot distributions, we removed plots with ten or fewer species in either the initial or final census, as adding or removing even a single species could produce extreme percentage changes (±10%).

After the selection process, our dataset compiled information from 406 plots (or grouped plots) covering ~420 ha (range 0.25–3 ha, mean plot size 1.04 ± 0.26 ha) with a cumulative monitoring time of 4,847 years (range 4.01–44.2, mean 11.94 ± 8.01 years). The earliest census dates were from 1971, and the latest were from 2021 (Supplementary Fig. 4).

### Regions

We divided the study area into six regions roughly following previous studies^61–64^. The division between the Northern and Central Andes was drawn at the border between Peru and Ecuador^64^. Supplementary Fig. 5 shows the relative floristic similarity of our plots and regions.

### Richness change

We calculated species richness as the number of fully identified species in each plot and census (SP). We calculated the change in species richness (% yr^−1^) as richness change = (((SP_initial_ − SP_final_)/SP_initial_) × 100)/time; where SP_initial_ and SP_final_ are the richness in the initial and final censuses, respectively, and time is the time interval between the initial and final censuses (in years). Palms (family Arecaceae) were included in the analyses (when included in both the initial and final censuses) as their exclusion did not have a significant effect on the results (Supplementary Fig. 6).

To test whether there was a significant change in richness through time, we used two-sided *t*-test analyses on richness change both for each of the regions independently and for the combined database. Given that the number of plots was unevenly distributed among the regions, to avoid sampling bias in the combined dataset analysis, we randomly sampled 30 plots per region and carried out a two-sided *t*-test with this subset. We repeated this process 1,000 times and obtained the averages of the *t*-test means and *P* values.

To assess potential linearity issues in the relationship between changes in the number of individuals and species richness, we calculated the change in species richness after rarefying both the initial and final censuses to the minimum number of individuals observed in either census (that is, whichever is lower) (package vegan). The correlation between the resulting rarefied richness change and the non-rarefied estimate (*r* = 0.74, *P* < 0.001) (Supplementary Fig. 7) supports the use of richness change and stem abundance change as independent variables (see ‘Predictor variables’ section) in the subsequent analyses.

Additional diversity indices and their change through time for each region were calculated using the vegan R package^65^ and tested in the same way as richness change (Supplementary Note 1).

#### Unidentified species and morphospecies

Despite the considerable identification efforts by all research groups involved in this project, many tree individuals remain unidentified (Indet indet), identified only to the genus level (for example, *Ocotea indet*) or classified as morphospecies (for example, *Ocotea* sp1, *Ocotea* sp2 and so on). These morphospecies codes were maintained through the multiple censuses and retroactively changed to a full species name in the database if one was given; however, the morphospecies criteria were not standardized across plots, nor were they curated. Because there is no obvious way to address these issues, we decided to (1) apply the restrictive selection criteria in terms of identification effort explained above, (2) exclude the unidentified individuals from the dataset, (4) use the genus-level information for the morphospecies (as their classification is not standardized across the dataset) and (4) exclude individuals identified only to the genus level from the species-level analyses. Consequently, some changes in species diversity are not captured due to these exclusions; however, we speculate that such unreported changes are probably caused by a small number of individuals that recruit or die without being identified across multiple censuses and are unlikely to be dominant members of the community.

To support the use of species-level data despite potential issues such as mistakes, changes in botanists and changes in the species concept through time, we calculated the change in genus richness in the same way as the change in species richness (but using the individual’s genus-level information, thus including morphospecies). Then, we calculated the correlation between the rate of change in proportional genus and species richness for the combined dataset (*r* = 0.711) (Supplementary Fig. 8) and for each region independently (Supplementary Table 9). Given the reasonably high correlation between the genus- and species-level richness change, we decided to continue working at the species level. Despite the challenges of working at this scale, we believe it was important to use this very valuable information and to try to address its shortcomings instead of reducing the available information by working at the genus level.

### Predictor variables

#### Baseline climate and climate change

To characterize the average climate and changing patterns for the Andes–Amazon area, we downloaded climatic data from TerraClimate^66^. We selected this product for its temporal resolution (monthly from 1958 to 2020), its spatial resolution (~4 km) and the availability of data for maximum temperature and annual precipitation. We used the ‘climateR’ package^67^ to download the TerraClimate monthly data from 1979 to 2020 (inclusive) for each of our plot locations based on their coordinates. We restricted the time series to post-1979 because of the higher uncertainty in earlier years. For each year, we used the monthly data to calculate the maximum ‘maximum temperature’ (°C), the sum of annual precipitation (mm) and the seasonality of precipitation (using monthly cumulative precipitation, coefficient of variation (CV) = 100 × (standard deviation/mean))^68^. For each plot, we used the data between 1979 and the year of its final census; for example, if the censuses are in 2000 and 2015, the climate variables provide information from 1979 to 2015. This way, we include any lagged effects of climate on forest dynamics, but we do not include post-census climate events that are not relevant in the database. For each plot, we estimated the mean values for the relevant time period to use as the baseline climate. For the same time period per plot, we performed a linear regression of the variable over time and used the slope as the annual rate of change. We show the relationship between the baseline value and the annual change for these variables (Supplementary Fig. 9). We also calculated the change of each variable in the complete 1979–2020 time period for reference.

#### Landscape context variables

To characterize the geography and structure of the area where each of the plots is located, we extracted elevation and landscape integrity from available datasets.

We downloaded and mosaiced the elevation rasters from the SRTM 90-m Digital Elevation Database v.4.1 from CGIAR-CSI^69^ and extracted the elevation values (m a.s.l.) for our plot locations.

We obtained tree cover data from the Global Forest Cover Change (GFCC) Tree Cover Multi-Year Global 30-m resolution raster^70^ via Google Earth Engine^71^. Tree cover is expressed as the percentage of pixel area covered by trees in 2015 (0–100%). We calculated the mean landscape integrity (%) as the mean tree cover for a radius of 50 km around each of the plot locations.

#### Structural variables

We calculated stem abundance change as the annual rate of proportional change in tree abundance per plot. To calculate this, we first computed the number of live individuals for each plot and census. To calculate the change in the number of individuals, we subtracted the initial from the final number of individuals, divided by the initial number of individuals, and multiplied by 100. Then, to calculate the annual rate of change in the proportional number of individuals, we divided this number by the time elapsed between the censuses. Due to species accumulation curves, this variable is crucial in determining richness change, and, as such, it is treated as an endogenous variable in the SEM.

We calculated the mortality rate (% yr^−1^) per plot using the ref. ^72^ equation together with the ref. ^73^ interval-length correction:

$$\mu =(((\mathrm{ln}({n}_{0})-\,\mathrm{ln}({n}_{{\rm{s}}}))/t)\times 100)\times {t}^{0.08}{,}$$

where *n*_0_ is the number of stems at the start of the census interval, *n*_s_ is the number of stems that survive that interval and *t* is the census interval length.

#### Sampling variables

To account for a potential change in the identification effort (for example, a large increase in individuals identified to genus level only), we calculated the change in the percentage of individuals per plot that were identified to species level in each plot (that is, the change in the percentage of identified individuals). We also included the time frame (years) between the initial and final censuses as a sampling variable. We show the total change in species through time per plot in Supplementary Fig. 10.

Further descriptors of each variable in each region can be found in Extended Data Fig. 2 and Supplementary Table 8.

### Regressions

To investigate the relationship between the predictors and richness change separately for the entire Andes–Amazon area, we performed linear regressions between each of the variables specified above (Table 1) (including census time frame) and the richness change per plot (annual rate of percentage change in richness) for the combined dataset. We explored second-order polynomial relationships for all variables and compared them with linear regressions using analysies of variance. Only temperature change (%) had a better fit using polynomial regression. Mortality rate was log transformed to better fulfil linear model assumptions. To assess the potential interference of spatial bias in the dataset, we bootstrapped each individual linear regression 100 times using random sets of 30 plots per region at each time, and we compared the direction of the slopes and their significance with those obtained from the complete dataset.

Finally, we performed regression analysis with interacting climate variables and richness change. In all cases, model residuals were checked to verify the fulfilment of the linear model assumptions.

### SEM

To evaluate the effect of the multiple variables directly on the richness change and indirectly via their effect on the stem abundance change and mortality rate, we performed a multigroup piecewise SEM (piecewiseSEM package)^74,75^ where the regions were the groups. This analysis evaluates the relationships for the combined dataset and each region separately to constrain coefficients with homogeneous effects across regions, leaving the remaining variables to vary freely. The standardized coefficients referred to as the ‘effects’ of one variable on another should be interpreted as their relative influence on the mean of the response. We excluded elevation because of the high correlation with maximum temperature (Supplementary Fig. 11) as the piecewise framework is unable to integrate correlated errors in its estimates. The SEM was estimated using three component linear models whose response variables were (1) mortality rate, (2) stem abundance change and (3) species richness change (Supplementary Fig. 12). We tested for normality, heteroscedasticity and the variable importance factor of each of the three component models to verify that the model assumptions were met, and that the inclusion of other variables with moderate correlation (Supplementary Fig. 11) did not create multicollinearity issues (variance inflation factor <4). The structure accounted for both the direct effect of mortality rate on changes in species richness—reflecting its role as a disturbance force that opens space and provides light for recruitment—and the indirect effect of mortality through its influence on changes in stem abundance, acting as a demographic force. Mortality rate was untransformed in order to facilitate the interpretation of results. Changes in identification effort were included only as a predictor of richness change, but not of stem abundance change or mortality rate, as there is no causal connection between these variables—an observation supported by the directed separation tests (*P* > 0.05) automatically performed in the piecewise analyses (Supplementary Table 10). We maintained this relatively simple partitioning of indirect paths to balance intrinsic uncertainty, the number of predictor variables, and a reduced sample size per region when applying the multigroup approach.

To estimate the indirect effects for each predictor and region, we multiplied the standardized path coefficients for each significant path (for example, maximum temperature → stem abundance change → richness change), considering paths via stem abundance change, mortality rate, and the longer combined path through stem abundance change and mortality rate. We computed these indirect effects only when each path coefficient was significant (*P* ≤ 0.05). Then, we added the indirect effects obtained by the three potential pathways and added them to estimate the total indirect effect of each predictor on richness change for each region. The direct effects are the standardized coefficients for the path between each predictor and the richness change for each region.

### Reporting summary

Further information on research design is available in the Nature Portfolio Reporting Summary linked to this article.

## Supplementary information

Supplementary Information Supplementary Tables 1–10, Figs. 1–12 and Notes 1 and 2.

Reporting Summary

## Source data

Source Data Fig. 1 Data to produce figure.

Source Data Fig. 2 Data to produce figure.

Source Data Fig. 3 Data to produce figure.

Source Data Fig. 4 Data to produce figure.

Source Data Fig. 5 Data to produce figure.

Source Data Extended Data Fig. 1 Data to produce figure.

Source Data Extended Data Fig. 2 Data to produce figure.

## Data Availability

The datasets generated and analysed within this study are owned and managed by many co-authors. Data are available from the corresponding author on reasonable request and with permission of relevant data owners. For more information, visit https://forestnet.com/ and https://www.missouribotanicalgarden.org/plant-science/plant-science/south-america/the-madidi-project/. Source data are provided with this paper.
