# Author Correction: Tree diversity is changing across tropical Andean and Amazonian forests in response to global change

**DOI:** 10.1038/s41559-026-03019-z

**Published:** 2026-02-20

**Authors:** B. Fadrique, F. Costa, F. Cuesta, G. Arellano, L. Cayuela, T. R. Baker, F. C. Draper, A. Esquivel-Muelbert, H. ter Steege, M. Bauters, J. Aguirre-Gutiérrez, Z. Aguirre-Mendoza, M. N. Alexiades, E. Alvarez-Davila, E. Arets, E. Ayala, C. G. A. Aymard, F. Baccaro, S. Báez, C. Baraloto, R. I. Barbosa, P. Barbosa Camargo, J. Barlow, P. E. Barni, J. Barroso, M. Benchimol, A. C. Bennett, E. Berenguer, L. Blanc, D. Bonal, F. Bongers, R. Brienen, F. Brown, M. BT Andrade, B. Burban, R. J. Burnham, J. L. Camargo, S. P. C. Carvalho, C. Castilho, J. Chave, F. Coelho de Souza, J. Comiskey, L. da Costa, R. B. de Lima, E. A. de Oliveira, R. L. C. de Oliveira, R. de Oliveira Perdiz, J. De Rutte, J. del Aguila-Pasquel, G. Derroire, A. Di Fiore, M. Disney, A. Duque, T. Emilio, W. Farfan-Rios, S. Fauset, P. M. Fearnside, K. J. Feeley, T. R. Feldpausch, J. Ferreira, L. Ferreira, G. R. Flores Llampazo, D. Galbraith, K. García-Cabrera, M. García Criado, E. Gloor, J. M. Grandez-Rios, B. Hérault, J. Homeier, E. N. Honorio Coronado, I. Huamantupa-Chuquimaco, W. Huaraca Huasco, Y. T. Huillca-Aedo, Á. Idárraga, O. Jadán-Maza, M. Kalamandeen, T. J. Killeen, S. G. W. Laurance, W. F. Laurance, A. Levesley, W. Lopez, M. J. Macía, W. E. Magnusson, Y. Malhi, A. G. Manzatto, B. S. Marimon, B. H. Marimon Junior, J. A. Martínez-Villa, M. B. Medeiros, K. Melgaço, L. Melo, T. Metzker, A. Monteagudo, P. S. Morandi, J. A. Myers, H. M. Nascimento, R. Nascimento, D. Neill, B. Nieto-Ariza, W. A. Palacios, S. Palacios-Ramos, N. C. Pallqui-Camacho, G. Pardo Molina, J. Peacock, M. A. Peña, R. T. Pennington, M. C. Peñuela, C. A. Peres, Á. J. Pérez, G. C. Pickavance, E. Pinto, J. Pipoly, N. Pitman, A. Prieto, H. Ramírez-Angulo, S. M. Reis, Z. Restrepo, C. Reynel, S. Ribeiro, G. Rivas-Torres, R. Rojas, A. Rudas, N. Salinas, R. P. Salomão, F. Santana, J. Schietti, G. Schwartz, J. Serrano, M. Silman, C. Silva, C. A. Silva, R. C. Silva, R. S. A. Silva, J. Silva-Espejo, M. Silveira, M. F. Simon, Y. C. Soto-Shareva, P. F. Souza, D. Storck-Tonon, J. Stropp, V. Swamy, J. S. Tello, J. Terborgh, R. Thomas, A. Torres-Lezama, J. D. Vale, L. Valenzuela Gamarra, G. van der Heijden, P. van der Hout, P. J. van der Meer, R. Vasquez Martinez, L. Vedovato, H. Verbeeck, I. Vieira, S. A. Vieira, E. Vilanova, B. Vinceti, V. A. Vos, R. Zagt, P. A. Zuidema, O. L. Phillips

**Affiliations:** 1 School of Environmental Sciences, University of Liverpool, Liverpool, UK; 2 School of Geography, University of Leeds, Leeds, UK; 3 Instituto Nacional de Pesquisas da Amazônia, Manaus, Brazil; 4 Global Research and Solutions Center, Universidad San Francisco de Quito, Quito, Ecuador; 5 Ecology and Evolutionary Biology, University of Michigan, Ann Arbor, MI USA; 6 Oikobit, Albuquerque, NM USA; 7 Instituto de Investigación en Cambio Global (IICG-URJC), Universidad Rey Juan Carlos, Móstoles, Spain; 8 Departamento de Biología y Geología, Física y Química Inorgánica, Universidad Rey Juan Carlos, Móstoles, Spain; 9 Department of Plant Sciences, University of Cambridge, Cambridge, UK; 10 School of Geography, Earth and Environmental Sciences, University of Birmingham, Birmingham, UK; 11 Naturalis Biodiversity Center, Leiden, the Netherlands; 12 Quantitative Biodiversity Dynamics, Department of Biology, Utrecht University, Utrecht, the Netherlands; 13 Q-ForestLab, Department of Environment, Ghent University, Ghent, Belgium; 14 Environmental Change Institute, School of Geography and the Environment, University of Oxford, Oxford, UK; 15 Leverhulme Centre for Nature Recovery, University of Oxford, Oxford, UK; 16 Universidad Nacional de Loja, Loja, Ecuador; 17 School of Anthropology and Conservation, University of Kent, Canterbury, UK; 18 Grupo de Investigación en Servicios Ecosistémicos y Cambio Climático, Fundación ConVida, Medellín, Colombia; 19 Wageningen Environmental Research, Wageningen University and Research, Wageningen, the Netherlands; 20 UNELLEZ-Guanare, Programa de Ciencias del Agro y el Mar, Herbario Universitario (PORT), Mesa de Caracas, Venezuela; 21 Jardín Botánico de Bogotá José Celestino Mutis, Bogotá, Colombia; 22 Departamento de Biologia, Universidade Federal do Amazonas, Manaus, Brazil; 23 Departamento de Biología, Escuela Politécnica Nacional del Ecuador, Quito, Ecuador; 24 MODEMAT Foundation for Mathematical Modeling and Education, Quito, Ecuador; 25 Institute of Environment, Florida International University, Miami, FL USA; 26 Instituto Nacional de Pesquisas da Amazônia (INPA - Núcleo de Roraima), Boa Vista, Brazil; 27 Centro de Energia Nuclear na Agricultura, Universidade de São Paulo, Piracicaba, Brazil; 28 Lancaster Environment Centre, Lancaster University, Lancaster, UK; 29 Roraima State University – UERR, Campus Rorainópolis, Rorainópolis, Brazil; 30 Universidade Federal do Acre, Centro Multidisciplinar, Cruzeiro do Sul, Brazil; 31 Applied Ecology and Conservation Lab, Universidade Estadual de Santa Cruz, Ilhéus, Brazil; 32 Forêts et Sociétés, Univ Montpellier, CIRAD, Montpellier, France; 33 INRAE, AgroParisTech, Université de Lorraine, Nancy, France; 34 Forest Ecology and Forest Management group, Wageningen University, Wageningen, the Netherlands; 35 Woodwell Climate Research Center, Falmouth, MA USA; 36 Federal University of Acre, Rio Branco, Brazil; 37 Independent Researcher, Manaus, Brazil; 38 INRAE, UMR ECOFOG, Kourou, French Guiana; 39 University Herbarium, University of Michigan, Ann Arbor, MI USA; 40 Rural University of Rio de Janeiro, Institute of Forestry, Seropedica, Brazil; 41 Centre de Recherche sur la Biodiversité et l’Environnement UMR5300, Université de Toulouse, CNRS, IRD, Toulouse, France; 42 BeZero Carbon, London, UK; 43 National Park Service, Fredericksburg, VA USA; 44 Smithsonian Institution, Washington, DC USA; 45 Instituto de Geociências, Universidade Federal do Pará, Belém, Brazil; 46 Universidade do Estado do Amapá, Amapá, Brazil; 47 Programa de Pós-Graduação em Ecologia e Conservação, Campus de Nova Xavantina, Universidade do Estado de Mato Grosso, Nova Xavantina, Brazil; 48 Universidade Estadual de Roraima, Boa Vista, Brazil; 49 Programa de pós-graduação em Recursos Naturais (PRONAT), Universidade Federal de Roraima, Boa Vista, Brazil; 50 Facultad de Ciencias Forestales, Universidad Nacional Agraria La Molina, Lima, Peru; 51 Universidad Nacional de la Amazonia Peruana, Iquitos, Peru; 52 Instituto de Investigaciones de la Amazonía Peruana, Iquitos, Peru; 53 Department of Forestry, Campus Darcy Ribeiro, University of Brasilia, Brasília, Brazil; 54 CIRAD, UMR EcoFoG (AgroParistech, CNRS, INRAE, Université des Antilles, Université de la Guyane), Kourou, French Guiana; 55 Cirad, UPR Forêts et Sociétés, University of Montpellier, Montpellier, France; 56 Department of Anthropology, The University of Texas at Austin, Austin, TX USA; 57 Estación de Biodiversidad Tiputini, Universidad San Francisco de Quito, Quito, Ecuador; 58 Department of Geography, University College London and NERC National Centre for Earth Observation, London, UK; 59 Departamento de Ciencias Forestales, Universidad Nacional de Colombia Sede Medellín, Medellín, Colombia; 60 Center for Research on Biodiversity Dynamics and Climate Change (CBioClima), Institute of Biosciences, São Paulo State University (UNESP), Rio Claro, Brazil; 61 Andrew Sabin Center for Environment and Sustainability and Department of Biology, Wake Forest University, Winston-Salem, NC USA; 62 Instituto Científico, Universidad Andina del Cusco, Cusco, Peru; 63 School of Geography, Earth and Environmental Sciences, University of Plymouth, Plymouth, UK; 64 Biology Department, University of Miami, Coral Gables, USA; 65 Faculty of Environment, Science and Economy, University of Exeter, Exeter, UK; 66 Embrapa Amazônia Oriental, Belém, Brazil; 67 Museu Paraense Emílio Goeldi, Belém-Pará, Brazil; 68 Universidad Nacional de San Antonio Abad del Cusco, Cusco, Peru; 69 School of Geosciences, The University of Edinburgh, Edinburgh, UK; 70 CREAF, Bellaterra (Cerdanyola del Vallès), Spain; 71 Conservation Ecology, University of Marburg, Marburg, Germany; 72 Resource Management, HAWK University of Applied Sciences and Arts, Goettingen, Germany; 73 Royal Botanic Gardens, Kew, Richmond, London, UK; 74 Herbario Alwyn Gentry, Departamento Académico de Ciencias Básicas, Universidad Nacional Amazónica de Madre de Dios, Madre de Dios, Peru; 75 Centro Ecológico INKAMAZONIA, Valle de Kosñipata, Cusco, Peru; 76 Department of Ecology and Environmental Science, Umeå University, Umeå, Sweden; 77 Asociación para la Investigación Tropical, Cusco, Peru; 78 Jadín Botánico de Medellín, Medellín, Colombia; 79 Grupo de Ecología Forestal y Agroecosistemas, Facultad de Ciencias Agropecuarias, Universidad de Cuenca, Campus Yanuncay, Cuenca, Ecuador; 80 Unique Land Use GmbH, Freiburg im Breisgau, Germany; 81 Museo de Historia Natural Noel Kempff Mercado, Santa Cruz de la Sierra, Bolivia; 82 Centre for Tropical Environmental and Sustainability Science, and College of Science and Engineering, James Cook University, Cairns, Queensland Australia; 83 Coltree, Medellín, Colombia; 84 Universidad Nacional de Colombia, Bogotá, Colombia; 85 Departamento de Biología, Área de Botánica, Universidad Autónoma de Madrid, Madrid, Spain; 86 Centro de Investigación en Biodiversidad y Cambio Global, Universidad Autónoma de Madrid, Madrid, Spain; 87 Departamento de Biologia, Universidade Federal de Rondônia, Porto Velho, Brazil; 88 Université du Quebec a Montreal, Montreal, Canada; 89 Embrapa Recursos Genéticos e Biotecnologia, Parque Estação Biológica, Prédio da Botânica e Ecologia, Brasilia, Brazil; 90 Universidade Federal do Oeste do Pará, Santarém, Brazil; 91 IBAM - Instituto Bem Ambiental, Belo Horizonte, Brazil; 92 Grupo MYR ESG solutions, Belo Horizonte, Brazil; 93 Washington University in St. Louis, St. Louis, MO USA; 94 Coordenação de Biodiversidade, Instituto Nacional de Pesquisas da Amazônia, Manaus, Brazil; 95 Instituto de Ciências Biológicas, Programa de Pós-Graduação em Ecologia, Universidade Federal do Pará, Belem, Brazil; 96 Universidad Estatal Amazónica, Facultad de Ingeniería Ambiental, Puyo, Ecuador; 97 Department of Wetland Ecology, Karlsruhe Institute of Technology, Karlsruhe, Germany; 98 Instituto Nacional de Biodiversidad, Quito, Ecuador; 99 Universidad Técnica del Norte, Ibarra, Ecuador; 100 Systematic and Evolutionary Botany Laboratory, Department of Biology, Ghent University, Gent, Belgium; 101 Instituto de Investigaciones Forestales de la Amazonía, Universidad Autónoma del Beni José Ballivián, Riberalta, Bolivia; 102 Department of Biology, Washington University, St. Louis, MO USA; 103 Geography, College of Life and Environmental Sciences, University of Exeter, Exeter, UK; 104 Grupo de Ecosistemas Tropicales y Cambio Global, Universidad Regional Amazónica Ikiam, Tena, Ecuador; 105 School of Environmental Sciences, University of East Anglia, Norwich, UK; 106 Center for Biodiversity and Global Change, Yale University, New Haven, CT USA; 107 Herbario QCA, Escuela de Ciencias Biológicas, Pontificia Universidad Católica de Ecuador, Quito, Ecuador; 108 Department of Biological Sciences, Auburn University, Auburn, AL USA; 109 Broward County Parks and Recreation, Oakland Park, FL USA; 110 Biological Sciences, Florida Atlantic University, Boca Raton, FL USA; 111 Collections, Conservation and Research, Field Museum of Natural History, Chicago, IL USA; 112 Instituto de Ciencias Naturales, Universidad Nacional de Colombia, Bogota, Colombia; 113 Facultad de Ciencias Forestales y Ambientales, Universidad de los Andes, Merida, Venezuela; 114 Centro de Ciências Biológicas e da Natureza, Universidade Federal do Acre, Rio Branco, Brazil; 115 Servicios Ecosistémicos y Cambio Climático, Fundación Con Vida and Corporación COL-TREE, Medellín, Colombia; 116 Universidad San Francisco de Quito, Estación de Biodiversidad Tiputini, Quito, Ecuador; 117 Wildlife Ecology and Conservation, University of Florida, Gainesville, FL USA; 118 Jardín Botánico de Missouri, Oxapampa, Pasco, Peru; 119 Instituto de Ciencias Naturales, Universidad Nacional de Colombia, Bogotá, Colombia; 120 Department of Science, Chemistry Section, Institute for Nature Earth and Energy, Pontifical Catholic University of Peru, Lima, Peru; 121 Programa de Pós-graduação em Ciências Biológicas, Universidade Federal Rural da Amazônia UFRA/CAPES, Belem, Brazil; 122 Universidade Federal do Amazonas, Manaus, Brazil; 123 Instituto de Pesquisa Ambiental da Amazônia, Brasília, Brazil; 124 Universidade do Estado de Mato Grosso, Caceres, Brazil; 125 Instituto Federal de Educação, Ciência e Tecnologia do Acre, Campus Baixada do Sol, Rio Branco, Brazil; 126 Universidad de La Serena, La Serena, Chile; 127 Instituto de Ecología y Biodiversidad, Santiago, Chile; 128 Department of Biogeography, Trier University, Trier, Germany; 129 Missouri Botanical Garden, St. Louis, MO USA; 130 Department of Biology, University of Florida, Gainesville, FL USA; 131 School of Science and Engineering, James Cook University, Cairns, Queensland Australia; 132 Iwokrama International Centre for Rain Forest Conservation and Development, Georgetown, Guyana; 133 Institute of Research for Forestry Development, Universidad de los Andes, Merida, Venezuela; 134 JDV Ambiental, Toledo, Brazil; 135 Herbario Selva Central HOXA, Oxapampa, Peru; 136 School of Geography, Univeristy of Nottingham, Nottingham, UK; 137 Form International, Hattem, the Netherlands; 138 Van Hall Larenstein University of Applied Sciences, Velp, the Netherlands; 139 Departament of Forest Sciences, University of São Paulo, São Paulo, Brazil; 140 Environmental Studies and Research Center, Universidade Estadual de Campinas, Campinas, Brazil; 141 VERRA, Washington, DC USA; 142 Bioversity International, Rome, Italy; 143 Tropenbos International, Ede, the Netherlands

**Keywords:** Climate-change ecology, Biodiversity, Macroecology

Correction to: *Nature Ecology & Evolution* 10.1038/s41559-025-02956-5, published online 23 January 2026.

In the version of the article initially published, in the Fig. 3a key, the blue triangle was labelled “–1.95 to <0” but should have been “<0 to 1.95” and the red triangle was labelled “>0 to 3.3” but should have been “<0 to –3.3”. These corrections have been made to the HTML and PDF versions of the article.

