# Supplementary material for: Tree diversity is changing across tropical Andean and Amazonian forests in response to global change
Source: Nat Ecol Evol. 2026 Jan 23;10(2):267–80. doi: 10.1038/s41559-025-02956-5 (PMC12890586; doi:10.1038/s41559-025-02956-5)
Supplement: Supplementary file 1 — Supplementary Tables 1–10, Figs. 1–12 and Notes 1 and 2. [file 41559_2025_2956_MOESM1_ESM.pdf]

nature ecology & evolution

Supplementary information

https://doi.org/10.1038/s41559-025-02956-5

Tree diversity is changing across tropical Andean and Amazonian forests in response to global change

In the format provided by the authors and unedited

SUPPLEMENTARY INFORMATION

Table of Contents

Supplementary Tables 2

Supplementary Figures... 18

Supplementary Notes 1 30

Supplementary Notes 2... 28

Supplementary Tables

Supplementary Table 1. Results from the bivariate regressions between richness change and the set of predictors.

Category	Predictor	Adj. R2	Statistic	p.value	Slope	Slope2
Baseline climate	Maximum Temperature	0.009	4.666	0.031	-0.020	
	Annual Precipitation	0.009	4.531	0.034	0.000	
	Precipitation seasonality	0.015	7.371	0.007	-0.007	
Climate change	Temperature change	0.035	8.259	0.000	3.478	-163.11
	Precipitation change	-0.002	0.063	0.802	-0.001	
	Precipitation seasonality change	0.027	12.182	0.001	-0.394	
Landscape context	Landscape integrity	0.100	45.851	0.000	0.272	
	Elevation	-0.001	0.538	0.464	-0.055	
Structure	Stem abundance change	0.007	3.847	0.05	0.005	
	Mortality rate (log)	0.014	6.759	0.010	0.000	
Sampling	Identification effort change	0.072	32.486	0.000	0.061	
	Time frame	-0.001	0.779	0.378	0.004	

Supplementary Table 2. Summary of the results from the bootstrapped bivariate regressions between richness change and the different predictors. Number of bootstrapped runs (total n=100) with positive or negative slopes and their significance for each predictor.

Category	Predictor	Positive		Negative	
		p.val≤0.05	p.val>0.05	p.val≤0.05	p.val>0.05
Baseline climate	Maximum Temperature	0	0	84	16
	Annual Precipitation	41	59	0	0
	Precipitation seasonality	0	0	90	10
Climate change	Temperature Change	0	0	82	18
	Precipitation change	0	48	0	52
	Prec seasonality change	0	0	57	43
Landscape context	Landscape integrity	0	84	0	16
	Elevation	92	8	0	0
Structure	Stem abundance change	100	0	0	0
	Mortality rate (log)	0	9	0	91
Sampling	Identification effort change	86	14	0	0
	Time frame	0	25	0	75

Supplementary Table 3. Regression results from all combinations of interacting climate variables (predictor 1 and 2) and richness change for the combined dataset. Adjusted R2 for the linear model with interaction, p value of that model, and p value of the interactive component within the model.

Predictor 1	Predictor 2	Adj. R2	p.value	Interaction p.value
Maximum Temperature	Temperature Change (oC/year)	0.021	**0.010**	0.084
Temperature Change (oC/year)	Annual Precipitation	0.032	**0.001**	0.135
Annual Precipitation	Precipitation change (mm/year)	0.008	0.105	0.801
Precipitation change (mm/year)	Precipitation seasonality	0.013	**0.043**	
**Precipitation seasonality**	**Prec seasonality change (cv/year)**	0.058	**0.000**	**0.012**
Maximum Temperature	Annual Precipitation	0.037	**0.000**	0.599
Temperature Change (oC/year)	Precipitation change (mm/year)	0.013	**0.044**	0.820
**Annual Precipitation**	**Precipitation seasonality**	0.025	**0.004**	**0.016**
Precipitation change (mm/year)	Prec seasonality change (cv/year)	0.030	**0.002**	0.075
Maximum Temperature	Precipitation change (mm/year)	0.005	0.160	
Temperature Change (oC/year)	Precipitation seasonality	0.034	**0.001**	0.965
**Annual Precipitation**	**Prec seasonality change (cv/year)**	0.048	**0.000**	**0.006**
Maximum Temperature	Precipitation seasonality	0.030	**0.002**	0.292
Temperature Change (oC/year)	Prec seasonality change (cv/year)	0.043	**0.000**	0.321
Maximum Temperature	Prec seasonality change (cv/year)	0.040	**0.000**	0.966

Supplementary Table 4. Diversity change indices mean and p values resulting from the two-sided t-test analyses for each region separately. See **Supplementary Notes 1** for index calculations.

		**Northern Andes**	**Central Andes**	**Western Amazon**	**Guyana Shield**	**Central-Eastern Amazon**	**Southern Amazon**
Richness	mean	**0.790**	**-0.200**	**0.150**	**-0.170**	**-0.200**	-0.150
	p	0.000	0.000	0.030	0.040	0.000	0.290
	t.statistic	3.618	-2.907	2.234	-2.149	-4.409	-1.064
Shannon	mean	**0.600**	**-0.340**	0.270	**-0.320**	**-0.410**	**-0.430**
	p	0.040	0.040	0.090	0.030	0.000	0.040
	t.statistic	2.184	-2.068	1.712	-2.248	-3.812	-2.113
Simpson	mean	0.070	**-0.260**	0.100	**-0.190**	**-0.340**	**-0.390**
	p	0.620	0.030	0.240	0.030	0.000	0.020
	t.statistic	0.499	-2.153	1.182	-2.186	-4.915	-2.529
Fisher	mean	**1.130**	**-0.220**	**0.260**	**-0.170**	**-0.180**	-0.050
	p	0.000	0.010	0.000	0.070	0.000	0.780
	t.statistic	3.892	-2.817	2.926	-1.876	-3.287	-0.281
Genus	mean	**0.340**	-0.090	-0.010	-0.130	**-0.160**	**-0.390**
	p	0.010	0.190	0.880	0.110	0.000	0.010
	t.statistic	2.719	-1.320	-0.151	-1.622	-3.854	-2.929
	n	32	76	63	53	145	37
	degrees of freedom	31	75	62	52	144	36

Supplementary Table 5. All standardized estimates, standard errors and p. values from the piecewise structural equation model. Variables constrained to all regions indicated by a "c"

Region	Response	Predictor	Std. Estimate	Std. Error	P. Value	Constrained
Northern Andes	Richness change	Maximum Temperature	-0.168	0.013	0.001	c
Northern Andes	Richness change	Annual Precipitation	0.124	0.000	0.011	c
Northern Andes	Richness change	Precipitation seasonality	-0.800	0.017	0.020	
Northern Andes	Richness change	Temperature change	0.009	1.727	0.596	c
Northern Andes	Richness change	Precipitation change	0.011	0.049	0.976	
Northern Andes	Richness change	Prec seasonality change	-0.699	1.590	0.023	c
Northern Andes	Richness change	Stem abundance change	0.267	0.042	0.000	
Northern Andes	Richness change	Mortality rate	0.406	0.322	0.140	
Northern Andes	Richness change	Tree Cover	0.322	0.038	0.289	c
Northern Andes	Richness change	Identification effort change	0.143	0.010	0.000	
Northern Andes	Richness change	Time frame	-0.455	0.077	0.171	
Central Andes	Richness change	Maximum Temperature	-0.305	0.013	0.001	c
Central Andes	Richness change	Annual Precipitation	0.182	0.000	0.011	c
Central Andes	Richness change	Precipitation seasonality	-0.757	0.018	0.007	
Central Andes	Richness change	Temperature change	0.025	1.727	0.596	c
Central Andes	Richness change	Precipitation change	0.142	0.044	0.420	
Central Andes	Richness change	Prec seasonality change	0.547	0.443	0.042	
Central Andes	Richness change	Stem abundance change	0.655	0.042	0.000	c
Central Andes	Richness change	Mortality rate	-0.313	0.059	0.014	
Central Andes	Richness change	Tree Cover	-0.461	0.009	0.019	
Central Andes	Richness change	Identification effort change	0.213	0.010	0.000	c
Central Andes	Richness change	Time frame	-0.025	0.021	0.829	
Western Amazon	Richness change	Maximum Temperature	-0.066	0.013	0.001	c
Western Amazon	Richness change	Annual Precipitation	0.246	0.000	0.011	c
Western Amazon	Richness change	Precipitation seasonality	0.006	0.008	0.974	
Western Amazon	Richness change	Temperature change	0.023	1.727	0.596	c
Western Amazon	Richness change	Precipitation change	-0.061	0.005	0.727	
Western Amazon	Richness change	Prec seasonality change	0.088	0.344	0.556	
Western Amazon	Richness change	Stem abundance change	0.310	0.042	0.000	c
Western Amazon	Richness change	Mortality rate	0.134	0.084	0.277	
Western Amazon	Richness change	Tree Cover	0.139	0.008	0.298	
Western Amazon	Richness change	Identification effort change	0.771	0.010	0.000	c
Western Amazon	Richness change	Time frame	-0.160	0.008	0.236	
Guyana Shield	Richness change	Maximum Temperature	-0.077	0.013	0.001	c
Guyana Shield	Richness change	Annual Precipitation	0.155	0.000	0.011	c
Guyana Shield	Richness change	Precipitation seasonality	-0.544	0.018	0.080	
Guyana Shield	Richness change	Temperature change	0.018	1.727	0.596	c
Guyana Shield	Richness change	Precipitation change	0.919	0.023	0.002	
Guyana Shield	Richness change	Prec seasonality change	0.153	1.772	0.491	
Guyana Shield	Richness change	Stem abundance change	0.429	0.042	0.000	c

Guyana Shield	Richness change	Mortality rate	-0.103	0.155	0.677	
Guyana Shield	Richness change	Tree Cover	0.280	0.017	0.208	
Guyana Shield	Richness change	Identification effort change	0.410	0.010	0.000	c
Guyana Shield	Richness change	Time frame	0.080	0.021	0.826	
Central-Eastern	Richness change	Maximum Temperature	-0.027	0.013	0.001	c
Central-Eastern	Richness change	Annual Precipitation	0.031	0.000	0.011	c
Central-Eastern	Richness change	Precipitation seasonality	0.198	0.011	0.147	
Central-Eastern	Richness change	Temperature change	0.028	1.727	0.596	c
Central-Eastern	Richness change	Precipitation change	-0.151	0.019	0.095	
Central-Eastern	Richness change	Prec seasonality change	-0.206	0.420	0.161	
Central-Eastern	Richness change	Stem abundance change	0.413	0.042	0.000	c
Central-Eastern	Richness change	Mortality rate	0.098	0.065	0.229	
Central-Eastern	Richness change	Tree Cover	0.111	0.005	0.205	
Central-Eastern	Richness change	Identification effort change	0.350	0.010	0.000	c
Central-Eastern	Richness change	Time frame	0.119	0.006	0.101	
Southern Amazon	Richness change	Maximum Temperature	-0.080	0.013	0.001	c
Southern Amazon	Richness change	Annual Precipitation	0.049	0.000	0.011	c
Southern Amazon	Richness change	Precipitation seasonality	0.841	0.032	0.007	
Southern Amazon	Richness change	Temperature change	0.019	1.727	0.596	c
Southern Amazon	Richness change	Precipitation change	-0.020	0.027	0.922	
Southern Amazon	Richness change	Prec seasonality change	-0.743	0.414	0.002	
Southern Amazon	Richness change	Stem abundance change	0.344	0.042	0.000	c
Southern Amazon	Richness change	Mortality rate	0.021	0.127	0.919	
Southern Amazon	Richness change	Tree Cover	0.982	0.017	0.007	
Southern Amazon	Richness change	Identification effort change	0.234	0.010	0.000	c
Southern Amazon	Richness change	Time frame	-0.023	0.025	0.913	
Northern Andes	Stem abundance	Maximum Temperature	-0.084	0.016	0.238	c
Northern Andes	Stem abundance	Annual Precipitation	0.000	0.000	0.737	c
Northern Andes	Stem abundance	Precipitation seasonality	0.361	0.011	0.175	
Northern Andes	Stem abundance	Temperature change	-0.052	2.059	0.045	c
Northern Andes	Stem abundance	Precipitation change	-0.039	0.034	0.897	
Northern Andes	Stem abundance	Prec seasonality change	0.049	0.124	0.081	c
Northern Andes	Stem abundance	Mortality rate	-0.729	0.158	0.000	
Northern Andes	Stem abundance	Tree Cover	0.167	0.026	0.521	
Northern Andes	Stem abundance	Time frame	0.063	0.005	0.018	c
Central Andes	Stem abundance	Maximum Temperature	-0.063	0.016	0.238	c
Central Andes	Stem abundance	Annual Precipitation	0.000	0.000	0.737	c
Central Andes	Stem abundance	Precipitation seasonality	-0.444	0.041	0.167	
Central Andes	Stem abundance	Temperature change	-0.058	2.059	0.045	c
Central Andes	Stem abundance	Precipitation change	0.025	0.103	0.906	
Central Andes	Stem abundance	Prec seasonality change	0.066	0.124	0.081	c
Central Andes	Stem abundance	Mortality rate	-0.423	0.131	0.003	
Central Andes	Stem abundance	Tree Cover	-0.209	0.021	0.356	
Central Andes	Stem abundance	Time frame	0.034	0.005	0.018	c
Western Amazon	Stem abundance	Maximum Temperature	-0.028	0.016	0.238	c

Western Amazon	Stem abundance	Annual Precipitation	0.000	0.000	0.737	c
Western Amazon	Stem abundance	Precipitation seasonality	0.076	0.007	0.640	
Western Amazon	Stem abundance	Temperature change	-0.113	2.059	0.045	c
Western Amazon	Stem abundance	Precipitation change	0.117	0.004	0.476	
Western Amazon	Stem abundance	Prec seasonality change	0.101	0.124	0.081	c
Western Amazon	Stem abundance	Mortality rate	-0.382	0.067	0.001	
Western Amazon	Stem abundance	Tree Cover	0.539	0.006	0.000	
Western Amazon	Stem abundance	Time frame	0.212	0.005	0.018	c
Guyana Shield	Stem abundance	Maximum Temperature	-0.024	0.016	0.238	c
Guyana Shield	Stem abundance	Annual Precipitation	0.000	0.000	0.737	c
Guyana Shield	Stem abundance	Precipitation seasonality	-0.170	0.014	0.365	
Guyana Shield	Stem abundance	Temperature change	-0.063	2.059	0.045	c
Guyana Shield	Stem abundance	Precipitation change	-0.091	0.018	0.597	
Guyana Shield	Stem abundance	Prec seasonality change	0.021	0.124	0.081	c
Guyana Shield	Stem abundance	Mortality rate	-0.811	0.072	0.000	
Guyana Shield	Stem abundance	Tree Cover	0.195	0.014	0.160	
Guyana Shield	Stem abundance	Time frame	0.165	0.005	0.018	c
Central-Eastern	Stem abundance	Maximum Temperature	-0.009	0.016	0.238	c
Central-Eastern	Stem abundance	Annual Precipitation	0.000	0.000	0.737	c
Central-Eastern	Stem abundance	Precipitation seasonality	-0.292	0.013	0.034	
Central-Eastern	Stem abundance	Temperature change	-0.103	2.059	0.045	c
Central-Eastern	Stem abundance	Precipitation change	0.345	0.022	0.000	
Central-Eastern	Stem abundance	Prec seasonality change	0.061	0.124	0.081	c
Central-Eastern	Stem abundance	Mortality rate	-0.672	0.060	0.000	
Central-Eastern	Stem abundance	Tree Cover	-0.083	0.006	0.350	
Central-Eastern	Stem abundance	Time frame	0.112	0.005	0.018	c
Southern Amazon	Stem abundance	Maximum Temperature	-0.031	0.016	0.238	c
Southern Amazon	Stem abundance	Annual Precipitation	-0.485	0.026	0.043	
Southern Amazon	Stem abundance	Precipitation seasonality	0.000	0.000	0.737	c
Southern Amazon	Stem abundance	Temperature change	-0.083	2.059	0.045	c
Southern Amazon	Stem abundance	Precipitation change	-0.323	0.022	0.057	
Southern Amazon	Stem abundance	Prec seasonality change	0.111	0.124	0.081	c
Southern Amazon	Stem abundance	Mortality rate	-0.575	0.088	0.000	
Southern Amazon	Stem abundance	Tree Cover	-0.239	0.015	0.408	
Southern Amazon	Stem abundance	Time frame	0.096	0.005	0.018	c
Northern Andes	Mortality rate	Maximum Temperature	0.148	0.023	0.141	c
Northern Andes	Mortality rate	Annual Precipitation	0.119	0.001	0.775	
Northern Andes	Mortality rate	Precipitation seasonality	0.519	0.006	0.000	c
Northern Andes	Mortality rate	Temperature change	0.226	38.268	0.632	
Northern Andes	Mortality rate	Precipitation change	-0.058	0.006	0.285	c
Northern Andes	Mortality rate	Prec seasonality change	0.478	1.306	0.106	
Northern Andes	Mortality rate	Tree Cover	-0.111	0.005	0.019	c
Northern Andes	Mortality rate	Time frame	-0.436	0.051	0.105	
Central Andes	Mortality rate	Maximum Temperature	0.106	0.023	0.141	c
Central Andes	Mortality rate	Annual Precipitation	0.545	0.000	0.004	

Central Andes	Mortality rate	Precipitation seasonality	-0.034	10.771	0.811	
Central Andes	Mortality rate	Temperature change	-0.013	0.006	0.285	c
Central Andes	Mortality rate	Precipitation change	0.163	0.006	0.000	c
Central Andes	Mortality rate	Prec seasonality change	0.547	0.912	0.039	
Central Andes	Mortality rate	Tree Cover	-0.119	0.005	0.019	c
Central Andes	Mortality rate	Time frame	0.368	0.041	0.001	
Western Amazon	Mortality rate	Maximum Temperature	0.033	0.023	0.141	c
Western Amazon	Mortality rate	Annual Precipitation	-0.107	0.000	0.614	
Western Amazon	Mortality rate	Precipitation seasonality	0.338	0.006	0.000	c
Western Amazon	Mortality rate	Temperature change	0.245	10.084	0.173	
Western Amazon	Mortality rate	Precipitation change	-0.164	0.006	0.285	c
Western Amazon	Mortality rate	Prec seasonality change	-0.067	0.608	0.714	
Western Amazon	Mortality rate	Tree Cover	-0.134	0.005	0.019	c
Western Amazon	Mortality rate	Time frame	0.117	0.014	0.461	
Guyana Shield	Mortality rate	Maximum Temperature	0.035	0.023	0.141	c
Guyana Shield	Mortality rate	Annual Precipitation	-0.862	0.001	0.037	
Guyana Shield	Mortality rate	Precipitation seasonality	0.243	0.006	0.000	c
Guyana Shield	Mortality rate	Temperature change	-0.822	19.805	0.002	
Guyana Shield	Mortality rate	Precipitation change	-0.052	0.006	0.285	c
Guyana Shield	Mortality rate	Prec seasonality change	0.627	2.692	0.005	
Guyana Shield	Mortality rate	Tree Cover	-0.096	0.005	0.019	c
Guyana Shield	Mortality rate	Time frame	-0.548	0.034	0.158	
Central-Eastern	Mortality rate	Maximum Temperature	0.016	0.023	0.141	c
Central-Eastern	Mortality rate	Annual Precipitation	0.072	0.001	0.466	
Central-Eastern	Mortality rate	Precipitation seasonality	0.229	0.006	0.000	c
Central-Eastern	Mortality rate	Temperature change	0.171	4.595	0.140	
Central-Eastern	Mortality rate	Precipitation change	-0.026	0.006	0.285	c
Central-Eastern	Mortality rate	Prec seasonality change	-0.247	0.752	0.247	
Central-Eastern	Mortality rate	Tree Cover	-0.186	0.005	0.019	c
Central-Eastern	Mortality rate	Time frame	0.183	0.011	0.068	
Southern Amazon	Mortality rate	Maximum Temperature	0.036	0.023	0.141	c
Southern Amazon	Mortality rate	Annual Precipitation	-0.374	0.002	0.292	
Southern Amazon	Mortality rate	Precipitation seasonality	0.126	0.006	0.000	c
Southern Amazon	Mortality rate	Temperature change	-0.024	25.001	0.940	
Southern Amazon	Mortality rate	Precipitation change	-0.031	0.006	0.285	c
Southern Amazon	Mortality rate	Prec seasonality change	0.242	0.771	0.345	
Southern Amazon	Mortality rate	Tree Cover	-0.141	0.005	0.019	c
Southern Amazon	Mortality rate	Time frame	-0.360	0.039	0.078	

Supplementary Table 6. Indirect standardised effects of each predictor variable for each region from the SEM multigroup analysis. There are three potential pathways of indirect effects for each predictor, via stem abundance change, via mortality rate and via the effect of mortality on stem abundance change.

**Region**	**Predictor**	**Indirect: Stem abundance change**		**Indirect: Mortality**		**Indirect: Mortality*Stem abundance change**	
		Estimate	sig	Estimate	sig	Estimate	sig
Northern Andes	Maximum Temperature	-0.023		0.060		-0.029	
	Annual Precipitation	0.000		0.048		-0.023	
	Precipitation seasonality	0.096		0.211		-0.101	***
	Temperature change	-0.014	*	0.092		-0.044	
	Precipitation change	-0.011		-0.024		0.011	
	Prec seasonality change	0.013		0.194		-0.093	
	Stem abundance change						
	Mortality rate	-0.194	***				
	Tree Cover	0.045		-0.045		0.022	*
	Identification effort change (%)						
	Time frame	0.017	*	-0.177		0.085	
Central Andes	Maximum Temperature	-0.041		-0.033		-0.029	
	Annual Precipitation	0.000		-0.171	**	-0.151	**
	Precipitation seasonality	-0.290		0.011		0.009	
	Temperature change	-0.038	*	0.004		0.004	
	Precipitation change	0.016		-0.051	***	-0.045	***
	Prec seasonality change	0.043		-0.171	*	-0.152	*
	Stem abundance change						
	Mortality rate	-0.277	**				
	Tree Cover	-0.137		0.037	*	0.033	*
	Identification effort change (%)						
	Time frame	0.022	*	-0.115	**	-0.102	**
Western Amazon	Maximum Temperature	-0.009		0.004		-0.004	
	Annual Precipitation	0.000		-0.014		0.013	
	Precipitation seasonality	0.024		0.045		-0.040	***
	Temperature change	-0.035	*	0.033		-0.029	
	Precipitation change	0.036		-0.022		0.019	
	Prec seasonality change	0.031		-0.009		0.008	
	Stem abundance change						
	Mortality rate	-0.118	***				
	Tree Cover	0.167	***	-0.018		0.016	*
	Identification effort change (%)						
	Time frame	0.066	*	0.016		-0.014	
Guyana Shield	Maximum Temperature	-0.010		-0.004		-0.012	
	Annual Precipitation	0.000		0.088		0.300	*
	Precipitation seasonality	-0.073		-0.025		-0.085	***

	Temperature change	-0.027	*	0.084	0.286	**
	Precipitation change	-0.039		0.005	0.018	
	Prec seasonality change	0.009		-0.064	-0.218	**
	Stem abundance change					
	Mortality rate	-0.348	***			
	Tree Cover	0.084		0.010	0.033	*
	Identification effort change (%)					
	Time frame	0.071	*	0.056	0.190	
Central-Eastern Amazon	Maximum Temperature	-0.004		0.002	-0.004	
	Annual Precipitation	0.000		0.007	-0.020	
	Precipitation seasonality	-0.121	*	0.022	-0.064	***
	Temperature change	-0.043	*	0.017	-0.048	
	Precipitation change	0.143	***	-0.003	0.007	
	Prec seasonality change	0.025		-0.024	0.069	
	Stem abundance change					
	Mortality rate	-0.278	***			
	Tree Cover	-0.034		-0.018	0.052	*
	Identification effort change (%)					
	Time frame	0.046	*	0.018	-0.051	
Southern Amazon	Maximum Temperature	-0.011		0.001	-0.007	
	Annual Precipitation	-0.167	*	-0.008	0.074	
	Precipitation seasonality	0.000		0.003	-0.025	***
	Temperature change	-0.029	*	-0.001	0.005	
	Precipitation change	-0.111		-0.001	0.006	
	Prec seasonality change	0.038		0.005	-0.048	
	Stem abundance change					
	Mortality rate	-0.198	***			
	Tree Cover	-0.082		-0.003	0.028	*
	Identification effort change (%)					
	Time frame	0.033	*	-0.008	0.071	

Supplementary Table 7. Direct, indirect, and total standardised effects of each predictor variable for each region from the SEM multigroup analysis.

Region	Predictor	Direct significant	Total indirect	Total significant
Northern Andes	Maximum Temperature	-0.168	0.000	-0.168
	Annual Precipitation	0.124	0.000	0.124
	Precipitation seasonality	-0.800	-0.101	-0.901
	Temperature change	0.000	-0.014	-0.014
	Precipitation change	0.000	0.000	0.000
	Prec seasonality change	-0.699	0.000	-0.699
	Stem abundance change	0.267	0.000	0.267
	Mortality rate	0.000	-0.194	-0.194
	Tree Cover	0.000	0.022	0.022
	Identification effort change (%)	0.143	0.000	0.143
	Time frame	0.000	0.017	0.017
Central Andes	Maximum Temperature	-0.305	0.000	-0.305
	Annual Precipitation	0.182	-0.322	-0.140
	Precipitation seasonality	-0.757	0.000	-0.757
	Temperature change	0.000	-0.038	-0.038
	Precipitation change	0.000	-0.096	-0.096
	Prec seasonality change	0.547	-0.323	0.224
	Stem abundance change	0.655	0.000	0.655
	Mortality rate	-0.313	-0.277	-0.590
	Tree Cover	-0.461	0.070	-0.391
	Identification effort change (%)	0.213	0.000	0.213
	Time frame	0.000	-0.195	-0.195
Western Amazon	Maximum Temperature	-0.066	0.000	-0.066
	Annual Precipitation	0.246	0.000	0.246
	Precipitation seasonality	0.000	-0.040	-0.040
	Temperature change	0.000	-0.035	-0.035
	Precipitation change	0.000	0.000	0.000
	Prec seasonality change	0.000	0.000	0.000
	Stem abundance change	0.310	0.000	0.310
	Mortality rate	0.000	-0.118	-0.118
	Tree Cover	0.000	0.183	0.183
	Identification effort change (%)	0.771	0.000	0.771
	Time frame	0.000	0.066	0.066
Guyana Shield	Maximum Temperature	-0.077	0.000	-0.077
	Annual Precipitation	0.155	0.300	0.455
	Precipitation seasonality	0.000	-0.085	-0.085
	Temperature change	0.000	0.259	0.259
	Precipitation change	0.919	0.000	0.919
	Prec seasonality change	0.000	-0.218	-0.218
	Stem abundance change	0.429	0.000	0.429

	Mortality rate	0.000	-0.348	-0.348
	Tree Cover	0.000	0.033	0.033
	Identification effort change (%)	0.410	0.000	0.410
	Time frame	0.000	0.071	0.071
Central-Eastern Amazon	Maximum Temperature	-0.027	0.000	-0.027
	Annual Precipitation	0.031	0.000	0.031
	Precipitation seasonality	0.000	-0.184	-0.184
	Temperature change	0.000	-0.043	-0.043
	Precipitation change	0.000	0.143	0.143
	Prec seasonality change	0.000	0.000	0.000
	Stem abundance change	0.413	0.000	0.413
	Mortality rate	0.000	-0.278	-0.278
	Tree Cover	0.000	0.052	0.052
	Identification effort change (%)	0.350	0.000	0.350
	Time frame	0.000	0.046	0.046
Southern Amazon	Maximum Temperature	-0.080	0.000	-0.080
	Annual Precipitation	0.049	-0.167	-0.118
	Precipitation seasonality	0.841	-0.025	0.816
	Temperature change	0.000	-0.029	-0.029
	Precipitation change	0.000	0.000	0.000
	Prec seasonality change	-0.743	0.000	-0.743
	Stem abundance change	0.344	0.000	0.344
	Mortality rate	0.000	-0.198	-0.198
	Tree Cover	0.982	0.028	1.010
	Identification effort change (%)	0.000	0.000	0.000
	Time frame	0.000	0.033	0.033

Supplementary Table 8. Descriptor statistics for the regional predictors calculated using individual plot data, i.e. Northern Andes mean Maximum temperature is the average of the combined maximum temperatures of the plots in that region. See Table 1 for predictors' information.

Category	Predictor	Descriptor	Northern Andes	Central Andes	Western Amazon	Guyana Shield	Central - Eastern Amazon	Southern Amazon
Baseline climate	Maximum temperature (ºC)	Mean	25.95	25.83	32.02	32.80	32.75	34.60
		SD	4.48	3.94	0.75	0.95	0.32	1.46
		Median	24.24	26.28	32.12	32.68	32.65	34.63
		Minimum	19.29	17.03	29.73	31.19	32.39	32.48
		Maximum	34.84	33.32	33.46	34.68	33.77	38.37
	Annual precipitation (mm)	Mean	2197.60	1392.34	2697.71	2363.48	2297.31	1685.32
		SD	766.18	542.67	647.11	444.28	84.24	208.04
		Median	2323.72	1307.50	2650.28	2171.62	2299.16	1654.01
		Minimum	822.63	455.67	1671.57	1538.31	1849.22	1150.74
		Maximum	3839.56	3307.25	4308.76	3406.15	2526.05	2206.21
	Precipitation seasonality (cv)	Mean	65.65	76.53	58.70	70.95	61.79	86.09
		SD	23.48	9.07	11.46	9.84	6.92	7.64
		Median	58.03	74.38	63.17	66.82	60.43	86.52
		Minimum	42.32	58.66	37.47	40.05	51.54	73.21
		Maximum	143.69	94.85	75.70	83.68	83.55	98.25
Climate change	Temperature change (ºC /year)	Mean	0.01	0.00	0.01	0.03	0.05	0.05
		SD	0.01	0.02	0.01	0.01	0.02	0.02
		Median	0.01	0.00	0.00	0.03	0.05	0.05
		Minimum	-0.04	-0.03	-0.05	0.00	-0.02	0.02
		Maximum	0.03	0.03	0.03	0.04	0.06	0.10
	Precipitation change (mm /year)	Mean	4.46	-3.18	5.22	3.57	3.76	-3.85
		SD	8.84	2.36	18.59	6.96	2.63	6.39
		Median	2.94	-2.67	-0.56	0.86	2.93	-3.96
		Minimum	-6.28	-7.17	-61.32	-6.91	-1.22	-16.28
		Maximum	42.89	-0.08	66.44	14.52	16.87	5.35
	Precipitation seasonality change (cv /year)	Mean	0.06	0.43	0.07	0.06	0.34	-0.08
		SD	0.22	0.36	0.23	0.07	0.19	0.45
		Median	0.03	0.33	0.07	0.04	0.37	0.10
		Minimum	-0.37	0.01	-0.49	-0.23	-0.24	-1.36
		Maximum	0.88	1.44	1.09	0.29	0.56	0.33
Landscape context	Landscape integrity (%)	Mean	41.42	51.34	71.08	56.23	62.63	43.12
		SD	9.62	12.64	8.70	7.42	10.74	16.47
		Median	41.46	52.18	72.56	55.00	57.04	46.23

		Minimum	17.10	25.80	36.25	35.87	34.94	18.87
		Maximum	68.55	75.44	78.84	76.92	80.88	64.81
	Elevation (m a.s.l.)	Mean	1533.25	1772.18	226.06	94.69	82.04	298.08
		SD	892.78	856.10	94.02	58.91	28.59	165.78
		Median	1870.25	1762.00	209.00	83.00	81.75	292.00
		Minimum	42.00	275.00	91.00	48.00	28.00	110.00
		Maximum	3198.50	3302.00	434.00	384.50	182.00	777.00
Structure	Stem abundance change (% /year)	Mean	0.00	-0.13	-0.03	-0.52	-0.11	-0.65
		SD	0.99	1.17	0.49	0.74	0.68	0.87
		Median	0.19	-0.09	0.04	-0.27	-0.09	-0.55
		Minimum	-2.63	-3.64	-1.64	-2.71	-3.77	-2.58
		Maximum	1.80	4.77	0.93	0.59	1.66	1.42
	Mortality rate (% /year)	Mean	2.34	2.95	2.83	1.85	1.69	3.58
		SD	1.01	1.25	0.76	0.91	0.68	1.36
		Median	2.02	2.84	2.66	1.70	1.56	3.35
		Minimum	1.14	0.93	1.61	0.36	0.48	1.78
		Maximum	6.67	7.86	6.05	4.86	5.60	7.09
Sampling	Identification effort change (%)	Mean	-1.68	-0.14	-0.48	1.08	-1.41	1.39
		SD	2.33	1.67	5.32	3.08	2.52	2.59
		Median	-0.79	0.00	0.00	0.00	-1.04	0.00
		Minimum	-8.85	-5.01	-14.79	-6.55	-13.36	-1.44
		Maximum	0.92	6.94	16.62	8.95	5.71	8.74
	Time frame (y)	Mean	8.08	9.25	20.89	13.67	9.54	12.48
		SD	5.15	3.31	8.57	10.08	6.32	6.91
		Median	7.08	8.92	22.04	11.25	6.05	9.12
		Minimum	4.01	4.37	4.86	4.01	4.87	4.32
		Maximum	22.60	15.06	37.87	44.20	28.45	24.41

Supplementary Table 9. Pearson's correlations (r) between species and genus level richness change per region.

Region	r	p.value
Northern Andes	0.552	0.001
Central Andes	0.702	0.000
Western Amazon	0.666	0.000
Guyana Shield	0.783	0.000
Central-Eastern Amazon	0.805	0.000
Southern Amazon	0.748	0.000

Supplementary Table 10- Results from the piecewise structural equation model Test of d-separation

Independ.Claim	Test.Type	DF	Crit.Value	P.Value
Stem abundance change ~ Identification effort change +	coef	395	-1.358	0.175
Moratlity rate ~ Identification effort change +	coef	396	0.536	0.592

Supplementary Figures

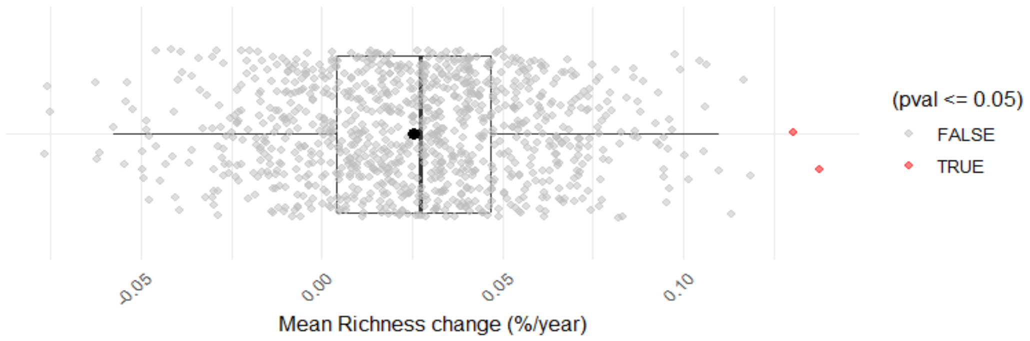

Supplementary Fig. 1. Mean results from the bootstrapped two-sided paired t-test testing the significance of the change in richness through time per plot, for the combination of 30 plots per region. Each point is one of the 1000 bootstrapped runs, if t test p value <=0.05 the point is red, otherwise grey. Error bars represent the most extreme data points, which are no more than 1.5 times quantiles 1 and 3 of the data (represented by the box limits). Box crossline represents the median. Black point represents mean.

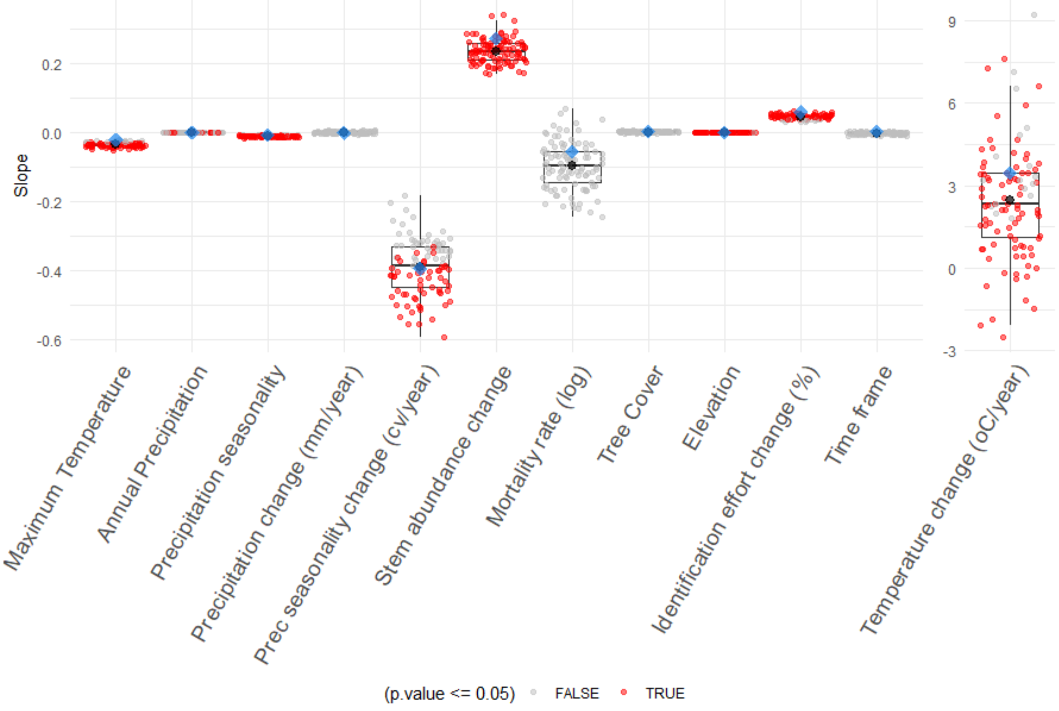

Supplementary Fig. 2. Slopes from the bootstrapped bivariate regressions between Richness change and the different predictors. Number of bootstrapped runs (n=100) with significant (p.value≤0.05) (red) or non-significant (grey) p values for each predictor. Slope from the complete dataset shown in **Fig.2** represented in blue. Note that the slope of temperature change is from the polynomial regression and on a different scale of the same units as y axis. Error bars represent the most extreme data points, which are no more than 1.5 times quantiles 1 and 3 of the data (represented by the box limits). Box crossline represents the median. Black point represents mean.

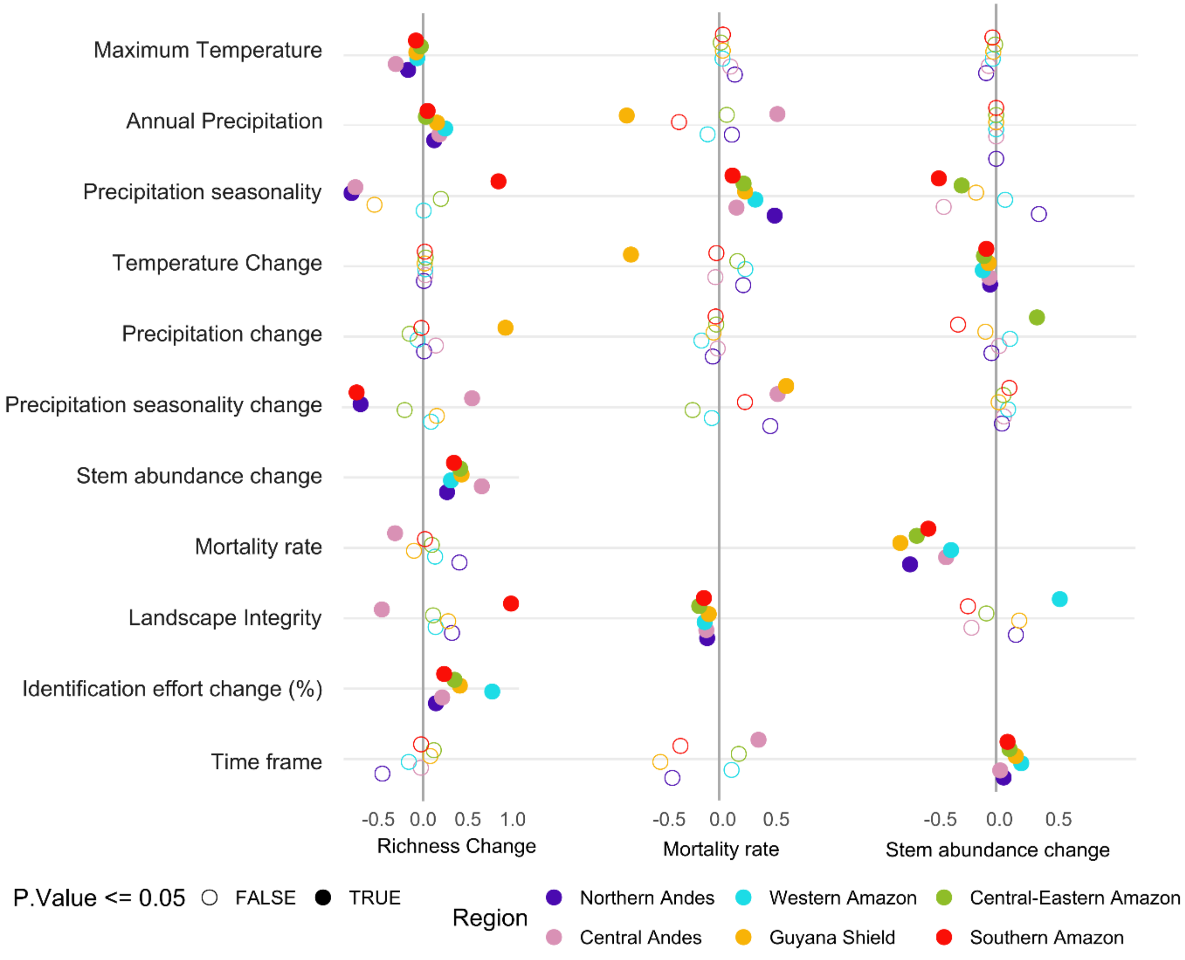

Supplementary Fig. 3 Standardised coefficients (effects) from the multigroup Structural Equation Model analysis for Richness change, Mortality rate, and Stem abundance change. Colours indicate regions; solid points indicate significant effects (p≤0.05) and hollow points indicate non-significant effects.

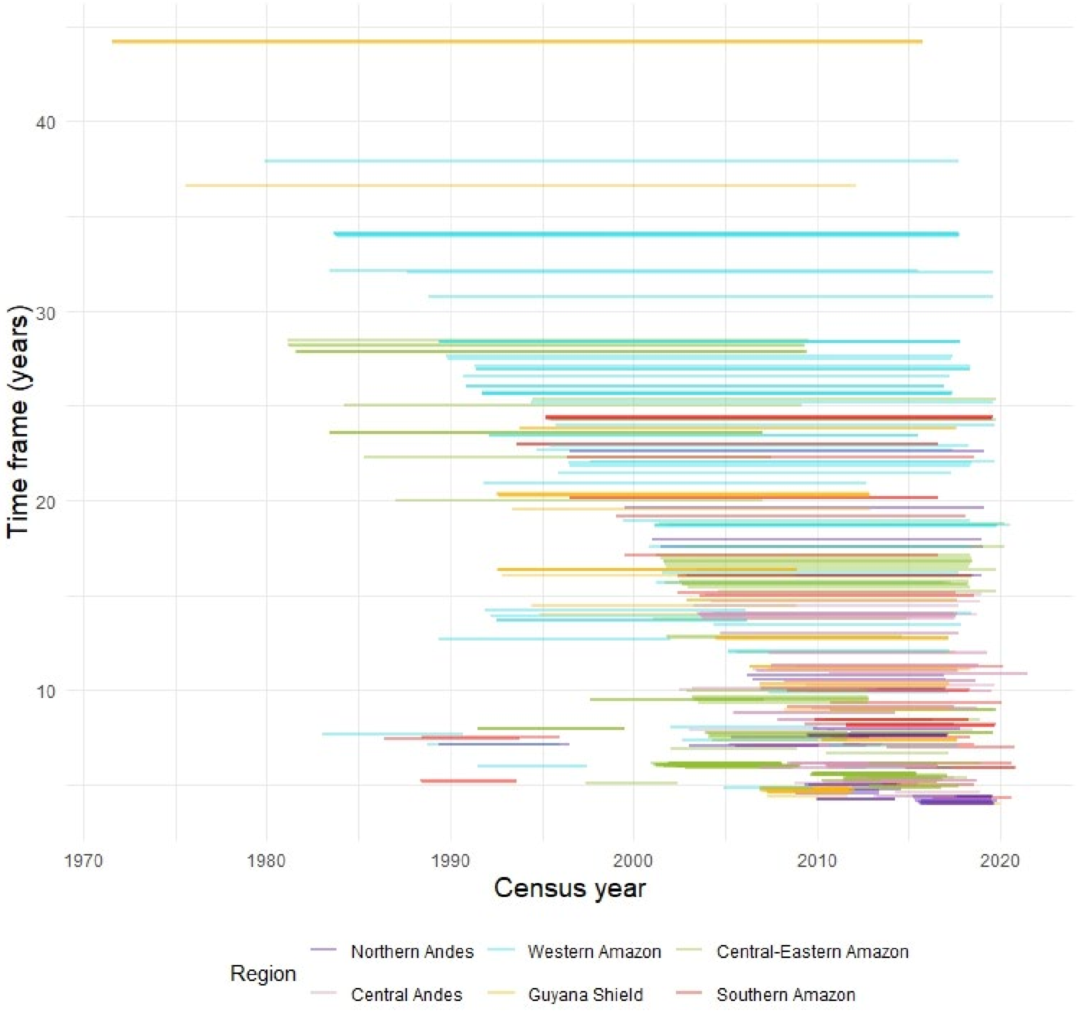

Supplementary Fig. 4. Researched census interval (duration vs initial and final census years) for each plot. Line colours correspond to regions.

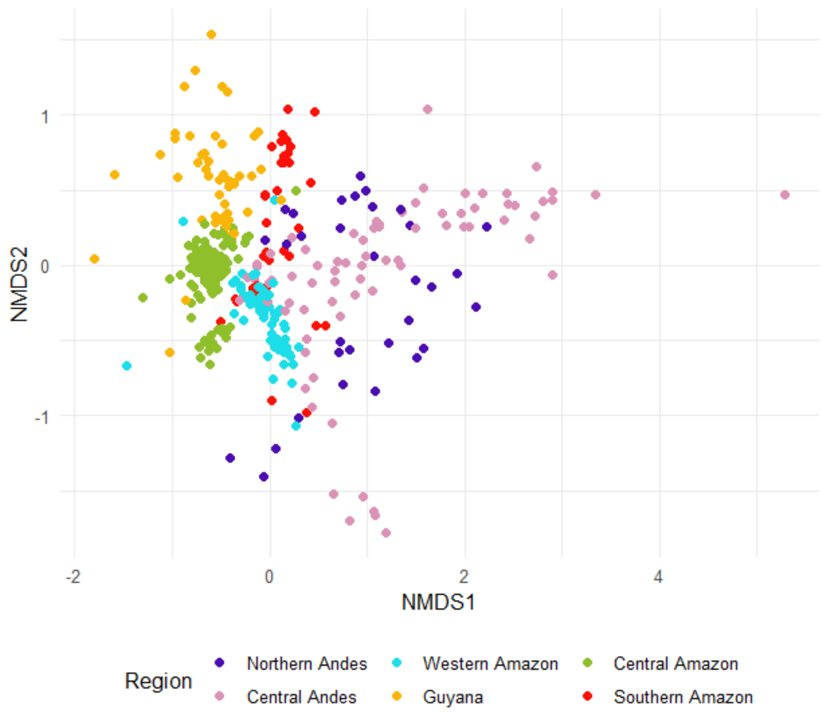

Supplementary Fig. 5. Non-metric multidimensional scaling (NMDS) (package "vegan") used to visualize the segregation of our plots in terms of compositional similarity. NMDS analyses were run on a Bray-Curtis dissimilarity matrix, excluding morphospecies, for at least 50 iterations and until a stable solution was reached (stress < 0.2). Each NMDS was optimised over two dimensions and displayed in an ordination plot.(n=406).

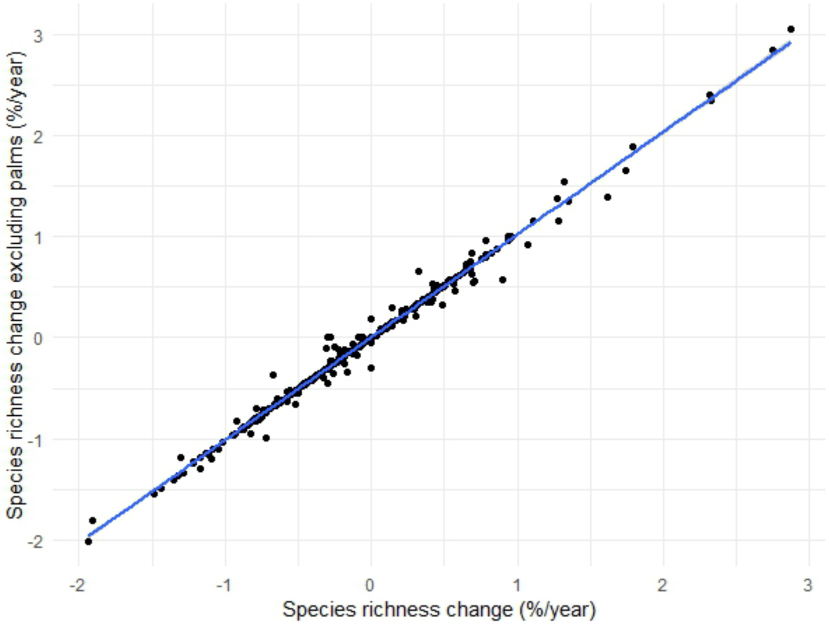

Supplementary Fig. 6. Pearson correlation between species richness change calculated excluding palm species (family Arecaceae) and species richness change with all the families included (R=0.995), only for plots with palms recorded (n=322). Each point represents a plot. Shaded ribbon represents 95% confidence interval.

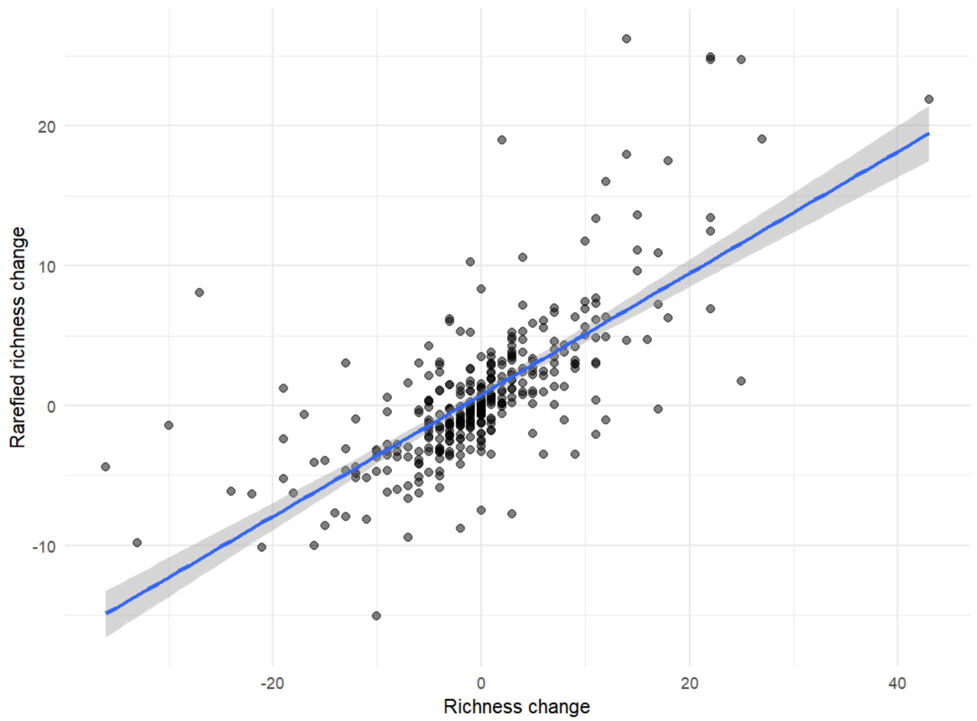

Supplementary Fig. 7. Pearson correlation between the rarefied species richness change (initial and final census rarefied using the lower number of individuals in these two censuses) and the richness change calculated as the difference in the number of species between the final and initial census ($r=0.74$, $n=406$). Each point represents a plot. Shaded ribbon represents 95% confidence interval.

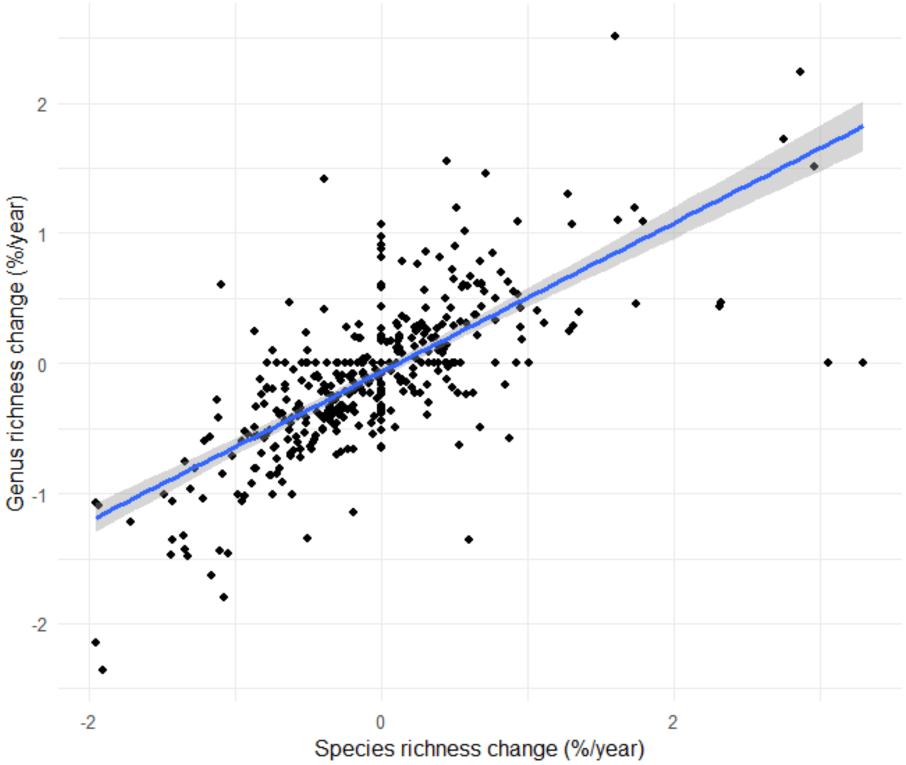

Supplementary Fig. 8. Pearson correlation between the change in genus richness (calculated in the same way as the change in species richness but including individuals only identified to Genus level) and the change in species richness for the combined dataset (r=0.711). Each point represents a plot. Shaded ribbon represents 95% confidence interval (n=406).

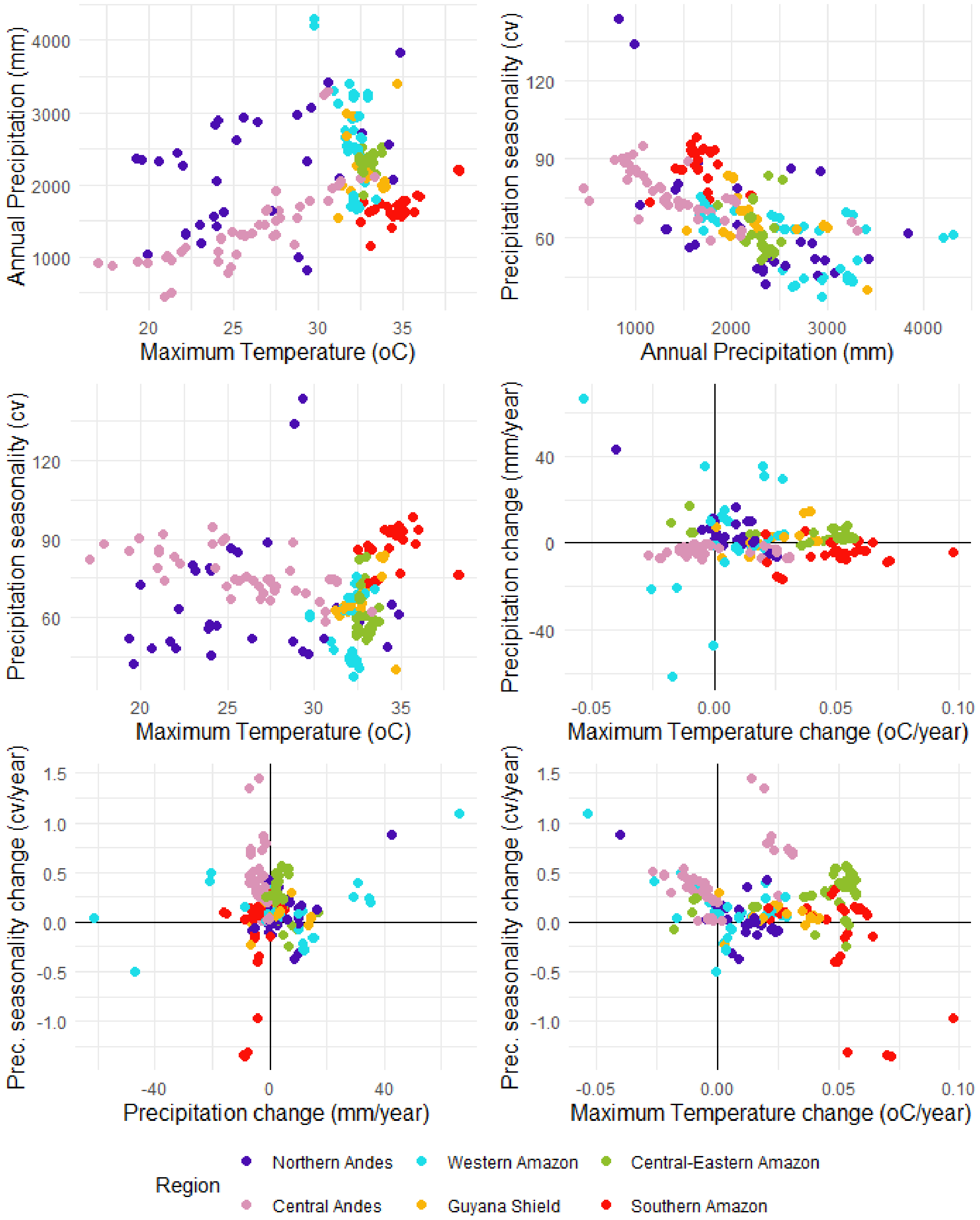

Supplementary Fig. 9. Relationship between the baseline climatic variables (maximum temperature, annual precipitation and precipitation seasonality), their change through time, and their proportional change through time. Each point represents a plot and the colour represents the region(n=406).

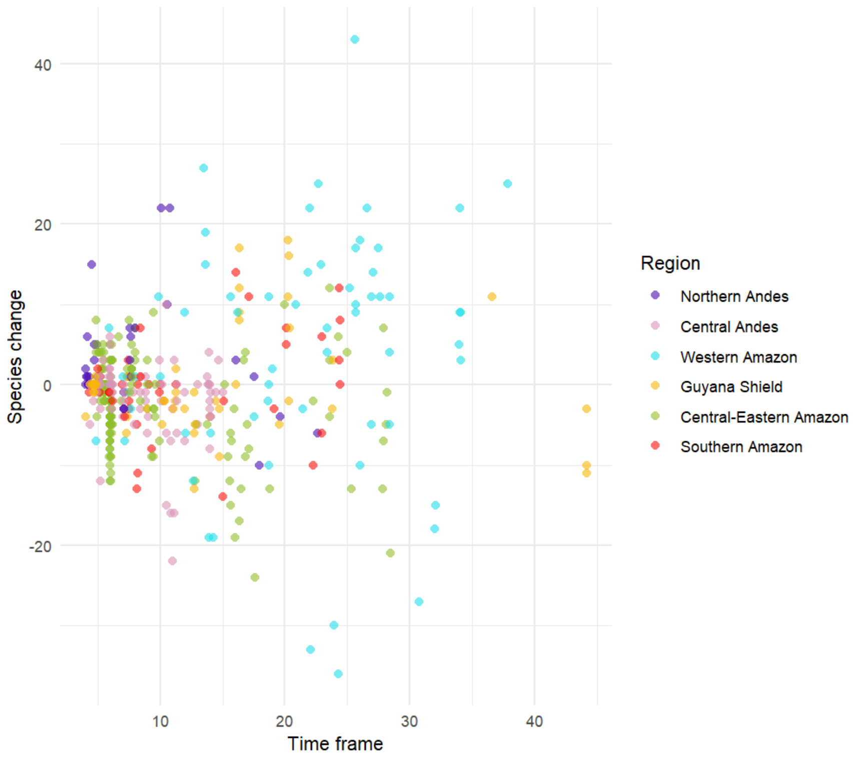

Supplementary Fig. 10. Species richness change (non-proportional) through time vs time elapsed between initial and final census. Each point represents a plot and the colour represents the region (n=406).

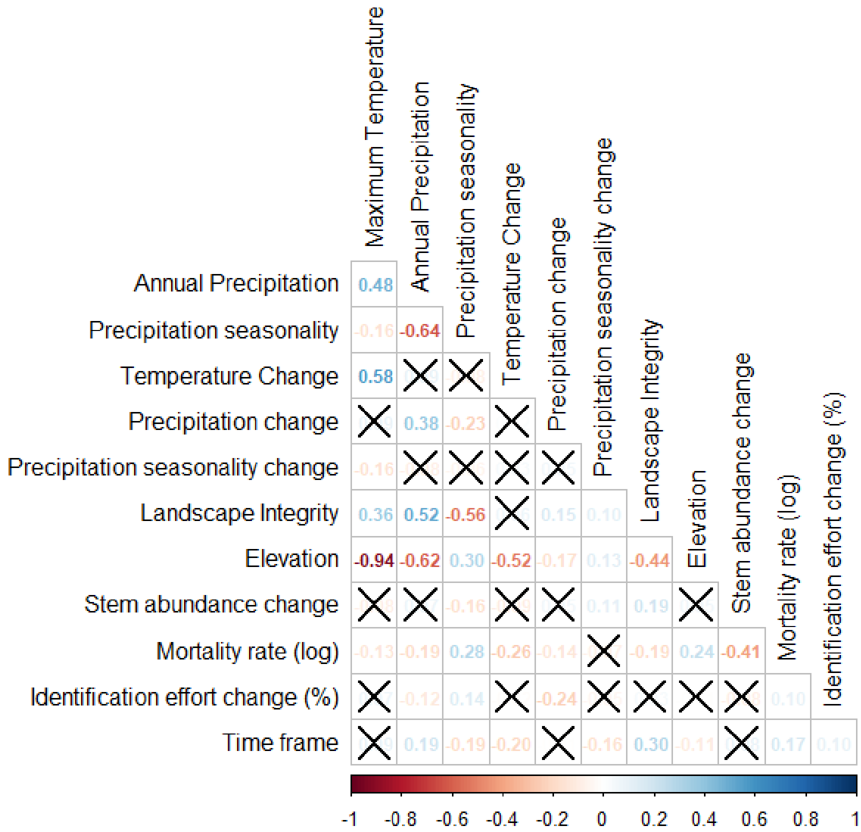

Supplementary Fig. 11. Correlations between predictors. Crosses mean non-significant correlations. Numbers indicate Pearson's r values. n=406

a)

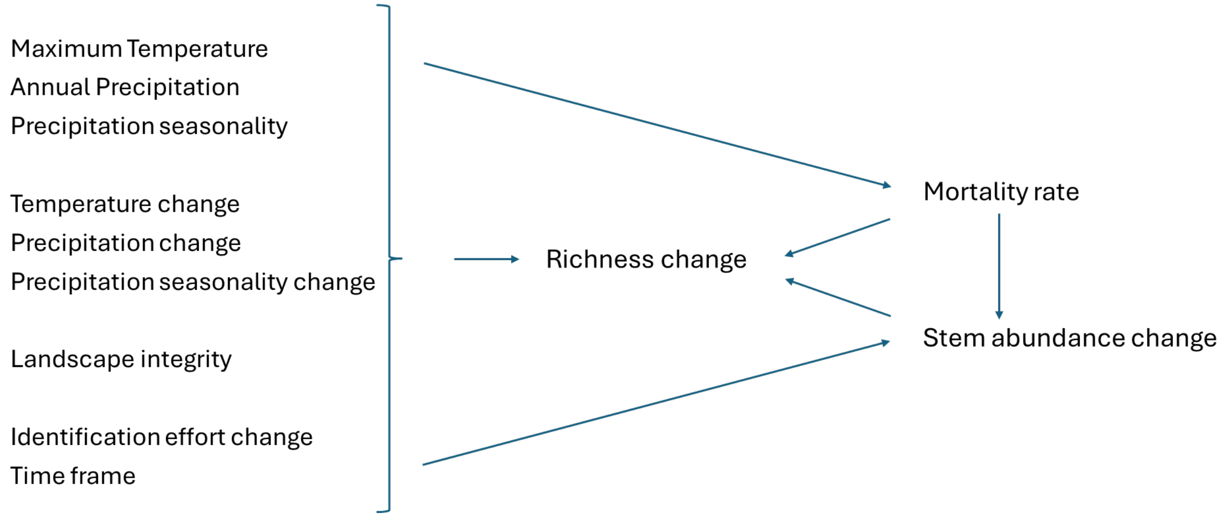

b)

```
PSEMmodel_<-psem(
lm(Stem abundance change ~
        Maximum Temperature + Annual Precipitation + Precipitation seasonality + Temperature change +
        Precipitation change +  Precipitation seasonality change + Mortality rate + Landscape integrity + Time frame,
        data_frame),
lm(Mortality rate ~
        Maximum Temperature + Annual Precipitation + Precipitation seasonality + Temperature change + Precipitation
        change +  Precipitation seasonality change + Landscape integrity + Time frame, data_frame),
lm(Richness change ~
        Maximum Temperature + Annual Precipitation + Precipitation seasonality + Temperature change +
        Precipitation change +  Precipitation seasonality change + Stem abundance change + Mortality rate +
        Landscape integrity + Identification effort change +  Time frame, data_frame),
data_frame)
```

Supplementary Fig. 12. Piecewise Structural Equation Model a) structure and b) script using package “piecewiseSEM”

Supplementary Notes

1. Diversity change indexes

Identifying and quantifying compositional change through time is challenging[1]. Multiple indices have been proposed to address this issue, however, it is unclear whether these could be applied to complicated datasets such as the one we present here, where the monitoring plots vary widely in composition, length of the study period, and beginning and end census dates. Thus, in addition to the richness change specified in the main text, we present here a combination of well-established diversity indices and their change through time. For some of them we converted the index to the effective number of species, and their change through time. The effective number of species (ENS) refers to the number of species in an equivalent community, with the same value of diversity index, composed of equally-abundant species; it is a way to make diversity indices more comparable and relatable[2]. The indices we looked at are 1) Species richness (on main text), which refers to the species count, it is a straightforward way to calculate and interpret changes in community composition; it is sensitive to rare species as it is based on presence/absence only. 2) Shannon diversity (ENS), considers the proportion of each species in the community and is equally sensitive to dominant and rare species. 3) Gini-Simpson diversity (ENS) is based on the probability that two randomly selected individuals belong to different species. It is more sensitive to abundant species while rare species have little impact on it. 4) Fisher's alpha, is less affected by the number of individuals. 5) Genus richness change, same as species richness but including all individuals identified to genus. 6) Sampling-corrected species richness change which considers the variation in identification effort change through the monitoring time.

For all calculations, we used the alive individuals in each plot and census excluding unidentified individuals and we looked at the proportional change in these indices over time. We used the R package "vegan"[3].

Shannon diversity

$$H \equiv -\sum_{i=1}^{S} p_i \ln p_i \qquad \text{ENS } H = \exp(H)$$

Shannon diversity change= (((ENS H initial-ENS H final)/ENS H initial)*100)/Time

We calculated the difference between final and initial Shannon effective number of species (ENS) and we divided this number by initial Shannon ENS to estimate percent change, then we divided by the time elapsed between the initial and final census to calculate the annual rate of percent change in Shannon ENS (%/year), to simplify the wording, we will refer to this variable as **Shannon diversity change.**

Gini-Simpsons diversity

We calculated the Simpsons diversity by converting Gini-Simpsons index to the ENS by applying the formula:

$GS \equiv 1-\sum_{i=1}^{S} p_i^2$ ENS GS: 1/(1-GS)

Simpson diversity change= (((ENS GS initial-ENS GS final)/ENS GS initial)*100)/Time

We calculated the difference between final and initial Simpsons diversity and divided it by the initial Simpsons diversity to obtain the percent change, then we divided it by the time elapsed between the initial and final census to calculate the annual rate of percent change in Simpson's diversity (%/year), to simplify the wording, we will refer to this variable as **Simpson diversity change.**

Fisher's alpha

S=*a**ln(1+*n*/*a*),

where *S* is the number of species, *n* is the number of individuals and *a* is Fisher's alpha.

Fisher's alpha change= (((*a* initial-*a* final)/*a* initial)*100)/Time

We calculated the difference between final and initial Fisher's alpha and divided it by the initial Fisher's alpha to obtain percent change and then by the time elapsed between the initial and final census to calculate the annual rate of percent change in Fisher's alpha (%/year), to simplify the wording, we will refer to this variable as **Fisher's alpha change.**

Genus

In order to support the use of species-level data despite its potential issues, (mistakes, changes in botanists, changes in the species concept through time, etc) we calculated the change in genus richness in the same way as the change in species richness to obtain the annual rate of percent change in genus richness (%/year).

Genus change= (((Genus initial-Genus final)/Genus initial)*100)/Time

Where Genus initial and Genus final are the genus richness in the initial and final censuses, respectively, and Time is the time interval between the initial and final censuses (in years).

Sampling-corrected richness change

To account for the effect of the change in the proportion of identified individuals in richness change, we calculated a sampling-corrected richness change. To do that we first explored regional regressions between the identification effort change and richness change and, when significant (Western Amazon, Central-Eastern Amazon, Guyana Shield), we used the regional slope as a correction factor for richness change.

$\text{Richness change corrected}_{(\text{regionX})} = \text{Richness change}_{(\text{regionX})} + (\text{Slope}_{(\text{regionX})} * \text{Identification effort change}_{(\text{regionX})})$

Changes in indices through time

To test whether the different diversity change indices indicated a significant difference from 0 (no change in diversity) we performed t-test analyses for each of the regions independently **(Table SI4).**

References

1. Hillebrand, H. *et al.* Biodiversity change is uncoupled from species richness trends: Consequences for conservation and monitoring. *Journal of Applied Ecology* **55**, 169–184 (2018).
2. Jost, L. Entropy and diversity. *Oikos* **113**, 363–375 (2006).
3. Oksanen, J. *et al.* vegan: Community Ecology Package. (2024).

2. Extended acknowledgements

This work was developed in part during the working group 'A Synthesis of Patterns and Mechanisms of Diversity and Forest Change in the Andes' funded by the Living Earth Collaborative at Washington University in St. Louis.

Among many contributors to this long-term forest ecology record, we acknowledge Alberto Vicentini, Alejandro Araujo-Murakami, Alexander Parada Gutierrez, Ana Andrade, Anand Roopsind, Anderson Pedro Bernardina Batista, Andrea Terán-Valdez, Antonio Peña Cruz, Antonella Bernardi, Antônio Tavares Mello, Atila Alves de Oliveira, Bert van Ulft, Carlos Quesada, Carolina Levis, Casimiro Mendoza, Claudinei Olivera dos Santos, Cinthia Pereira de Oliveira, Darcy Galiano Cabrera, Diego Armando Silva da Silva, Eduardo Hase, Eliana Jiménez, Everton Almeida, Fernando Cornejo Valverde, Freddy Ramirez Arevalo, Gabriela Lopez-Gonzalez, German Toasa, Hans Buttgenbach Verde, Iêda Leão do Amaral, Irina Mendoza Polo, Jadson Coelho de Abreu, James Richardson, Joey Talbot, Laura Cifuentes, Lidiany Carvalho, Lily Rodriguez Bayona, Lourens Poorter, Luciana de Oliveira Pereira, Luis Gustavo Canesi Ferreira, Luiz Aragão, Luzmila Arroyo, Marcelino Carneiro Guedes, Marcos Salgado Vital, Maria do Socorro, Marielos Peña-Claros, Massiel Corrales Medina, Mathias Tobler, Maxime Réjou-Méchain, Michel Baisie, Nikolay Aguirre, Olga Martha Montiel, Pedro Salcedo, Percy Núñez Vargas, Perseu da Silva Aparício, Pétrus Naisso, Rafael Herrera, Raimunda Oliveira de Araújo, Samaria Murakami, Thaiane Rodrigues de Sousa, Toby Gardner, Victor Chama Moscoso, Vincent Bezard, Wegliane Campelo da Silva and Wendeson Castro.
