# Supplementary material for: Tree diversity is changing across tropical Andean and Amazonian forests in response to global change
Source: Nat Ecol Evol. 2026 Jan 23;10(2):267–80. doi: 10.1038/s41559-025-02956-5 (PMC12890586; doi:10.1038/s41559-025-02956-5)
Supplement: Supplementary file 2 — Reporting Summary [file 41559_2025_2956_MOESM2_ESM.pdf]

nature portfolio

Corresponding author(s): Belen Fadrique

Last updated by author(s): Oct 30, 2025

Reporting Summary

Nature Portfolio wishes to improve the reproducibility of the work that we publish. This form provides structure for consistency and transparency in reporting. For further information on Nature Portfolio policies, see our Editorial Policies and the Editorial Policy Checklist.

Statistics

For all statistical analyses, confirm that the following items are present in the figure legend, table legend, main text, or Methods section.

n/a	Confirmed	
☐	☒	The exact sample size (n) for each experimental group/condition, given as a discrete number and unit of measurement
☐	☒	A statement on whether measurements were taken from distinct samples or whether the same sample was measured repeatedly
☐	☒	The statistical test(s) used AND whether they are one- or two-sided<br>*Only common tests should be described solely by name; describe more complex techniques in the Methods section.*
☐	☒	A description of all covariates tested
☐	☒	A description of any assumptions or corrections, such as tests of normality and adjustment for multiple comparisons
☐	☒	A full description of the statistical parameters including central tendency (e.g. means) or other basic estimates (e.g. regression coefficient) AND variation (e.g. standard deviation) or associated estimates of uncertainty (e.g. confidence intervals)
☐	☒	For null hypothesis testing, the test statistic (e.g. F, t, r) with confidence intervals, effect sizes, degrees of freedom and P value noted<br>*Give P values as exact values whenever suitable.*
☒	☐	For Bayesian analysis, information on the choice of priors and Markov chain Monte Carlo settings
☒	☐	For hierarchical and complex designs, identification of the appropriate level for tests and full reporting of outcomes
☐	☒	Estimates of effect sizes (e.g. Cohen's d, Pearson's r), indicating how they were calculated

Our web collection on statistics for biologists contains articles on many of the points above.

Software and code

Policy information about availability of computer code

Data collection	No code or software was use in data collection
Data analysis	Data analysis was primarily conducted using the open source programming language R version 4.2.0. The dplyr v.1.1.4, tidyr 1.3.1, data.table v.1.17.2 and reshape v.0.8.9 packages were used for data preparation. Package TNRS v.0.3.6 was used to standardize species names. Climate data was accessed via the climateR v.0.3.7 package. The vegan v.2.7.1 package was used to complete the NDMS ordination analysis and the diversity index calculations. Package piecewiseSEM v.2.1.2was used to conduct the multigroup piecewise structural equation model analyses. Figures were generated using the packages ggplot2 v.3.5.2, ggpubr v.0.6.0, corrplot v.0.95 and QGIS software version 3.30.0.

For manuscripts utilizing custom algorithms or software that are central to the research but not yet described in published literature, software must be made available to editors and reviewers. We strongly encourage code deposition in a community repository (e.g. GitHub). See the Nature Portfolio guidelines for submitting code & software for further information.

Data

Policy information about availability of data

All manuscripts must include a data availability statement. This statement should provide the following information, where applicable:

- Accession codes, unique identifiers, or web links for publicly available datasets
- A description of any restrictions on data availability
- For clinical datasets or third party data, please ensure that the statement adheres to our policy

The datasets generated and analysed within this study are owned/managed by many different co-authors. Data are available from the corresponding author on reasonable request and with permission of relevant data owners. For more information visit www.Forestplots.net

Research involving human participants, their data, or biological material

Policy information about studies with human participants or human data. See also policy information about sex, gender (identity/presentation), and sexual orientation and race, ethnicity and racism.

Reporting on sex and gender	*Use the terms sex (biological attribute) and gender (shaped by social and cultural circumstances) carefully in order to avoid confusing both terms. Indicate if findings apply to only one sex or gender; describe whether sex and gender were considered in study design; whether sex and/or gender was determined based on self-reporting or assigned and methods used. Provide in the source data disaggregated sex and gender data, where this information has been collected, and if consent has been obtained for sharing of individual-level data; provide overall numbers in this Reporting Summary. Please state if this information has not been collected. Report sex- and gender-based analyses where performed, justify reasons for lack of sex- and gender-based analysis.*
Reporting on race, ethnicity, or other socially relevant groupings	*Please specify the socially constructed or socially relevant categorization variable(s) used in your manuscript and explain why they were used. Please note that such variables should not be used as proxies for other socially constructed/relevant variables (for example, race or ethnicity should not be used as a proxy for socioeconomic status). Provide clear definitions of the relevant terms used, how they were provided (by the participants/respondents, the researchers, or third parties), and the method(s) used to classify people into the different categories (e.g. self-report, census or administrative data, social media data, etc.) Please provide details about how you controlled for confounding variables in your analyses.*
Population characteristics	*Describe the covariate-relevant population characteristics of the human research participants (e.g. age, genotypic information, past and current diagnosis and treatment categories). If you filled out the behavioural & social sciences study design questions and have nothing to add here, write "See above."*
Recruitment	*Describe how participants were recruited. Outline any potential self-selection bias or other biases that may be present and how these are likely to impact results.*
Ethics oversight	*Identify the organization(s) that approved the study protocol.*

Note that full information on the approval of the study protocol must also be provided in the manuscript.

Field-specific reporting

Please select the one below that is the best fit for your research. If you are not sure, read the appropriate sections before making your selection.

☐ Life sciences ☐ Behavioural & social sciences ☒ Ecological, evolutionary & environmental sciences

For a reference copy of the document with all sections, see nature.com/documents/nr-reporting-summary-flat.pdf

Ecological, evolutionary & environmental sciences study design

All studies must disclose on these points even when the disclosure is negative.

Study description	We assembled a dataset of 406 floristic inventory plots. Based on presence/absence (trees with dbh>10cm) and species level identifications, we calculate the species richness at two different points in time, and estimate the percent change in richness through time on each plot. We use that data to evaluate the overall trend in Andes-Amazon richness change and the trends in regional change (6 regions). We use climatic data (baseline and change), environmental data and structural data to understand the potential role of these variables in driving the change in richness. We first perform bivariate regressions with the complete dataset. We then perform a multigroup structural equation model to understand the effect of each factor on the richness change of each region.
Research sample	The sample consisted of 406 floristic inventory plots distributed across the Andes and Amazonia, each plot contained individual diameter measurements and species level identifications for woody plants >10 cm in diameter during two points in time, separated by at least 4 years. The sample was used to represent changes in species richness across the Andes-Amazon tree flora. Original data was collected by co-authors and their teams following similar field protocols, with the purpose of long-term forest structure and compositional monitoring.

Sampling strategy	We used as many inventory plots that fulfilled our criteria as were available across the tropical Andes and Amazon basin. Because the plots are not distributed evenly across the basin and because plots varied in size we used a spatially-stratified bootstrap resampling approach to ensure the dataset was sampled as evenly as possible when estimating richness change across the Andes-Amazon area. This approach is described in detail in the methods text, but briefly consisted of repeatedly sampling a standard number of plots (30) per region. Then for the regional analysis we used all plots available per region.
Data collection	Data was collected by coauthors and their teams. Collections consisted of standardized floristic inventory plots, where all individual trees had their diameter measured and identified to the highest possible taxonomic resolution. Data was uploaded and curated at Forestplots.org
Timing and spatial scale	Data was collected by coauthors over from the mid 1980's to present. The minimum time between census is 4 years (range= 4.01 – 44.2; mean=11.94+-8.01 years). The spatial scale is the tropical Andes and the entire Amazon (figure 2).
Data exclusions	All individual that could not be identified to species level were excluded from all analysis. In the methods we discussed extensively the steps taken to deal with unidentified individuals and those identified only to genus level (morphospecies).
Reproducibility	Data consist of observations and not experiments, therefore it was not relevant to reproduce findings.
Randomization	Samples groups were defined by geographical regions that are explicitly defined. We followed resampling procedures where plots were randomly selected to performed the Andes-Amazon (entire area) analysis.
Blinding	not relevant to this observational study design.

Did the study involve field work? ☒ Yes ☐ No

Field work, collection and transport

Field conditions	Fieldwork work was conducted across the tropical Andes and the Amazonian rainforest during different periods of time and therefore different climatic conditions. A description of the climatic variables at each region is provided on the SI
Location	Observational data collected across the tropical Andes and the Amazon in South America, ranging from -17 to 8.5 latitudinal degrees and -80 to -47 longitudinal degrees,
Access & import/export	For this project no exportation was required
Disturbance	No disturbance was caused

Reporting for specific materials, systems and methods

We require information from authors about some types of materials, experimental systems and methods used in many studies. Here, indicate whether each material, system or method listed is relevant to your study. If you are not sure if a list item applies to your research, read the appropriate section before selecting a response.

Materials & experimental systems

n/a	Involved in the study	
☒	☐	Antibodies
☒	☐	Eukaryotic cell lines
☒	☐	Palaeontology and archaeology
☒	☐	Animals and other organisms
☒	☐	Clinical data
☒	☐	Dual use research of concern
☒	☐	Plants

Methods

n/a	Involved in the study	
☒	☐	ChIP-seq
☒	☐	Flow cytometry
☒	☐	MRI-based neuroimaging

Plants

Seed stocks	*Report on the source of all seed stocks or other plant material used. If applicable, state the seed stock centre and catalogue number. If plant specimens were collected from the field, describe the collection location, date and sampling procedures.*
Novel plant genotypes	*Describe the methods by which all novel plant genotypes were produced. This includes those generated by transgenic approaches, gene editing, chemical/radiation-based mutagenesis and hybridization. For transgenic lines, describe the transformation method, the number of independent lines analyzed and the generation upon which experiments were performed. For gene-edited lines, describe the editor used, the endogenous sequence targeted for editing, the targeting guide RNA sequence (if applicable) and how the editor was applied.*
Authentication	*Describe any authentication procedures for each seed stock used or novel genotype generated. Describe any experiments used to assess the effect of a mutation and, where applicable, how potential secondary effects (e.g. second site T-DNA insertions, mosiacism, off-target gene editing) were examined.*
